# A Pathogenic Mechanism in Huntington's Disease Involves Small CAG-Repeated RNAs with Neurotoxic Activity

**DOI:** 10.1371/journal.pgen.1002481

**Published:** 2012-02-23

**Authors:** Mónica Bañez-Coronel, Silvia Porta, Birgit Kagerbauer, Elisabet Mateu-Huertas, Lorena Pantano, Isidre Ferrer, Manuel Guzmán, Xavier Estivill, Eulàlia Martí

**Affiliations:** 1 Genes and Disease Programme, Centre for Genomic Regulation (CRG) and Universitat Pompeu Fabra, Barcelona, Catalonia, Spain; 2 Network of Biomedical Research in Epidemiology and Public Health (CIBERESP), Madrid, Spain; 3 Institute of Neuropathology, IDIBELL-Bellvitge University Hospital, L'Hospitalet de Llobregat, Catalonia, Spain; 4 Network of Biomedical Research in Neurodegenerative Disorders (CIBERNED), Madrid, Spain; 5 Department of Biochemistry and Molecular Biology I, Complutense University, Madrid, Spain; The Hospital for Sick Children and University of Toronto, Canada

## Abstract

Huntington's disease (HD) is an autosomal dominantly inherited disorder caused by the expansion of CAG repeats in the Huntingtin *(HTT)* gene. The abnormally extended polyglutamine in the HTT protein encoded by the CAG repeats has toxic effects. Here, we provide evidence to support that the mutant *HTT* CAG repeats interfere with cell viability at the RNA level. In human neuronal cells, expanded *HTT* exon-1 mRNA with CAG repeat lengths above the threshold for complete penetrance (40 or greater) induced cell death and increased levels of small CAG-repeated RNAs (sCAGs), of ≈21 nucleotides in a Dicer-dependent manner. The severity of the toxic effect of *HTT* mRNA and sCAG generation correlated with CAG expansion length. Small RNAs obtained from cells expressing mutant *HTT* and from HD human brains significantly decreased neuronal viability, in an Ago2-dependent mechanism. In both cases, the use of anti-miRs specific for sCAGs efficiently blocked the toxic effect, supporting a key role of sCAGs in HTT-mediated toxicity. Luciferase-reporter assays showed that expanded HTT silences the expression of CTG-containing genes that are down-regulated in HD. These results suggest a possible link between HD and sCAG expression with an aberrant activation of the siRNA/miRNA gene silencing machinery, which may trigger a detrimental response. The identification of the specific cellular processes affected by sCAGs may provide insights into the pathogenic mechanisms underlying HD, offering opportunities to develop new therapeutic approaches.

## Author Summary

Huntington's disease (HD) is a neurodegenerative disorder caused by an abnormal CAG expansion in the Huntingtin gene (*HTT*), resulting in an expanded polyglutamine track in the HTT protein. Longer CAG expansions correlate with an earlier more severe manifestation of the disease that produces choreic movement, behavioural and psychiatric disturbances, and dementia. Although the causative gene is widely expressed, neuropathology is characterized by striatal and cortical atrophy. HTT interacts with proteins involved in transcription, cell signaling, and transport. The pathogenic role of mutant HTT is not fully understood. This study shows that CAG expanded HTT RNA also contributes to neuronal toxicity. Mutant HTT RNA gives rise to small CAG-repeated RNAs (sCAGs) with neurotoxic activity. These short RNAs interfere with cell functions by silencing the expression of genes that are fully or partially complementary, through a mechanism similar to that of microRNAs. These findings suggest that a small RNA–dependent mechanism may contribute to HD neuronal cell loss. The exhaustive identification of the target genes modulated by sCAGs may lead to a better understanding of HD pathology, allowing the development of new therapeutic strategies.

## Introduction

Huntington disease (HD), a dominantly inherited neurodegenerative disorder, is caused by an abnormal CAG expansion within the first exon of the Huntingtin gene (HTT), leading to an expanded polyglutamine (polyQ) track in the HTT protein. HTT is ubiquitously expressed in the cytoplasm of most cells in the body, with higher expression levels in brain and testis [1], [2]. However the disease shows a selective pattern of neurodegeneration, with clear effects in the cerebral cortex, and a more pronounced neuropathology in the striatum [3], [4].

The number of CAG repeats influences the severity and the age of onset of the disease. Longer expansions associate with a more severe form and an earlier manifestation of the disease [5].

It has been widely reported that the polyQ expansion in the HTT protein leads to protein aggregation and cell toxicity [6], a mechanism thought to be primarily involved in several neurological disorders caused by CAG repeats [7]–[10]. However, whether the mutant HTT aggregates are pathogenic, incidental or neuroprotective is still controversial. It has been shown that mutant HTT aggregates may function as sinks where essential proteins are sequestered [11], compromising cell survival [12]. Other studies show that increased levels of diffuse mutant HTT are responsible for neuronal cell death [13]. In agreement with the two possibilities, the activation of autophagy, reduce both soluble mutant protein and aggregate levels, and reduces toxicity [14], [15].

In addition to the widely described pathogenic role of expanded polyQ tracks, several studies have also shown that different neurodegenerative disorders caused by trinucleotide repeat expansions may involve RNA-mediated mechanisms [16], [17]. These include the sequestration of RNA-binding proteins by the expanded trinucleotide repeats, and activation of a variety of pathways such as RNA interference (RNAi) and protein misfolding pathways. The understanding of how expanded-repeat RNAs confer neurotoxicity is crucial to developing effective treatments.

A neurotoxic effect for CAG-expanded transcripts has been recently demonstrated in Drosophila models of Ataxin-3 [18] and Myotonic Dystrophy [19]. In the later, the authors propose a pathogenic role of siRNAs derived from complementary sense and anti-sense expanded (CUG/CAG) transcripts. In line with this, double-stranded CAG/CUG repeat RNA produced by bidirectional transcription induces neurodegeneration and movement disorder in Drosophila model [20]. This neurotoxic effect is largely dependent on Dicer activity and linked to the formation of (CAG)7mers. In addition, other studies describe that trinucleotide repeated transcripts form secondary structures [21] that can be cleaved by Dicer *in vitro* [22], [23] resulting in the generation of trinucleotide repeated short RNAs. Together, these data suggest that different mechanisms lead to the formation of aberrant small RNAs in trinucleotide expansion diseases.

Huntington's disease like 2 (HDL2) is caused by a CTG.CAG expansion in the JPH3 gene, and the neuropathologic outcome and clinical features largely resemble HD. CUG expansions in the JPH3 gene correlates both with the formation of RNA foci and cell toxicity, suggesting RNA mediated toxicity [24], [25]. RNA pathogenic mechanisms have been little explored in HD. Expanded HTT transcripts are retained in the nucleus of human HD fibroblasts and co-localize with the MBNL1 protein [26], a splicing factor involved in the pathogenesis of CTG/CAG expanded transcripts [27]. In addition, mutant HTT protein alters microRNA (miRNA) biogenesis [28], and a strong miRNA deregulation is observed in HD brains [29]–[32], which may contribute to the aberrant gene expression observed in HD.

Here we provide evidence for a pathogenic role of the mutant *HTT* RNA. CAG-expanded *HTT* RNA can be processed to generate CAG-repeated short RNAs with neurotoxic activity. We show that expanded HTT toxic effect is dependent on RNA-induced silencing complex (RISC) and further demonstrate that expanded HTT participates in posttranscriptional gene silencing of genes containing pure and interrupted CTG repeats. This, together with HTT polyglutamine toxicity, may contribute to the neurodegeneration pattern observed in HD.

## Results

### Expanded exon 1 of human *HTT* is toxic at the RNA level

**Figure 1:**
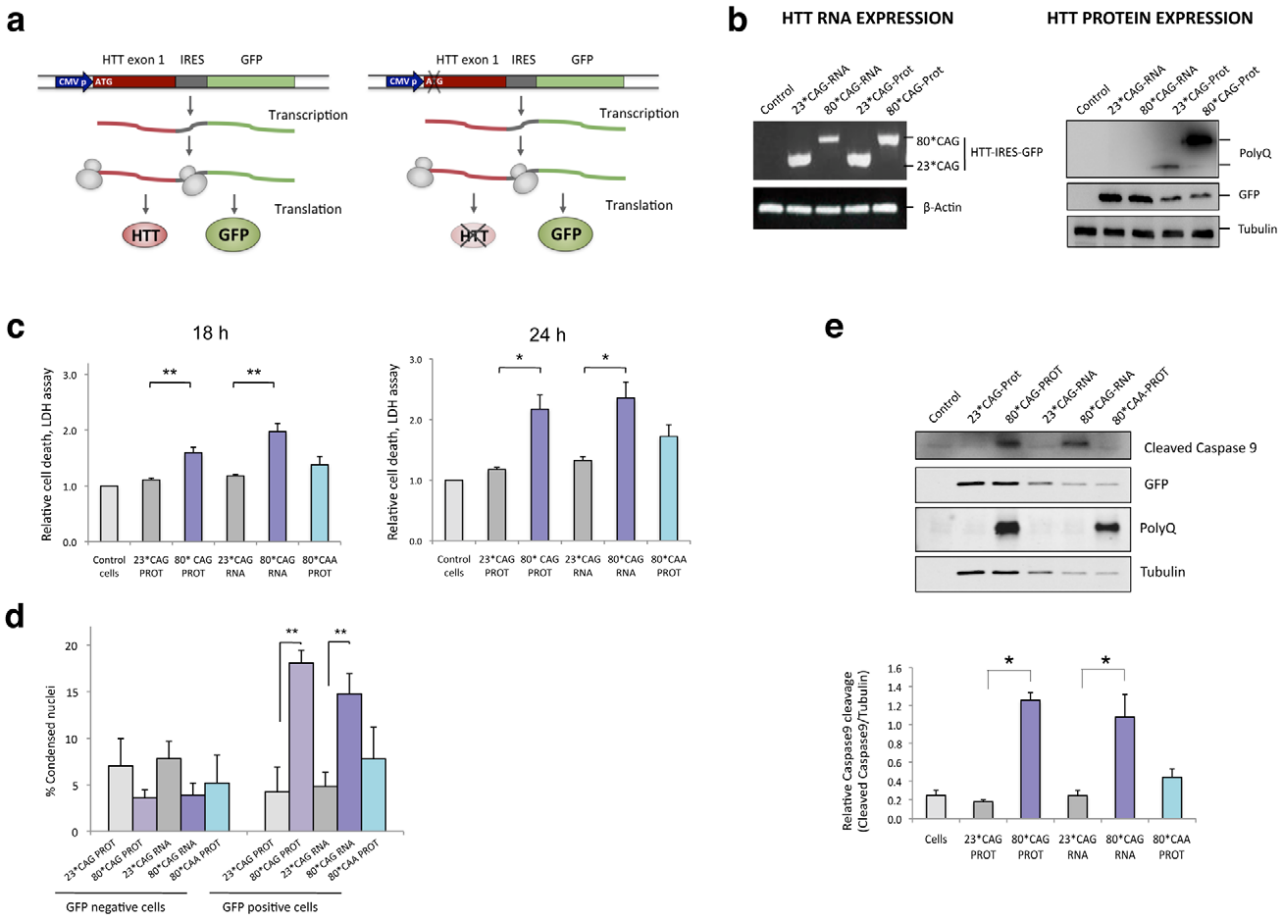
CAG-expanded exon 1 of human *HTT* is toxic at the RNA level. A. CAG-unexpanded (wild-type; 23 CAG repeats) and CAG-expanded (mutant; 80 CAG repeats) constructs of human *HTT* exon 1 (*HTT* exon 1) were subcloned into a pIRES-EGFP vector. Each variant was produced as a normal translated form (left) and a form lacking the translation initiation codon (right). The specific role of the expanded protein was analyzed with a construct expressing CAA-expanded HTT-e1. The use of IRES-based bicistronic vectors with a GFP reporter allows monitoring of transfected cells. B. The four different constructs express the mRNA *HTT*-IRES-GFP (left) and the GFP reporter protein (right). HTT protein is only expressed in the constructs containing the ATG translation initiation codon (right). C. Differentiated SH-SY5Y cells were transfected with the HTT-IRES-GFP vectors and LDH cell toxicity assay was performed 18 h and 24 h after transfection. Expression of CAG-expanded *HTT* (RNA or protein) resulted in dramatic cell death. CAA-expanded HTT-e1 didn't induce a significant effect on cell viability at the time points analyzed (n = 4; *p<0.05, **p<0.01, ***p<0.001). D. The percentage of dead transfected cells was also determined 36 hours after transfection by counting 200 GFP-negative cells (left) and 200 GFP-positive cells (right), scoring in each case the presence of nuclear fragmentation. Values represent the percentage of cells showing nuclear condensation in each situation ± SD (n = 3; **p<0.01). E. Expression of CAG-expanded *HTT* RNA induced caspase 9 cleavage. GFP blots highlight the expression of all constructs in transfected cells and polyglutamine (PolyQ) blots show expression of expanded HTT protein. Densitometry determinations of cleaved caspase 9 *vs.* α-Tubulin were performed on cells lysated 24 hours after transfection. Results are presented as the mean of arbitrary optical density units (O.D. units ± SEM; n = 3; *p<0.05, ***p<0.001). In C. and E., values represent the mean fold change with respect to the control non-transfected cells ± SEM.

To evaluate the contribution of CAG-expanded RNA in HD pathogenesis, we generated vectors expressing unexpanded and CAG-expanded forms of exon 1 of human *HTT* (HTT-e1). HTT-e1 constructs containing 23 CAG repeats (23*CAG) were used as wild-type (unexpanded) model. For the expanded HD mutation, we generated HTT-e1 constructs containing 80 CAG repeats (80*CAG). Each set of vectors was produced as a form that could be translated into protein, and as a variant lacking the translation initiation codon, that was only expressed as RNA. Due to the reduced size of HTT-e1, the different variants were cloned into a pIRES-GFP expression vector. This strategy allowed the monitoring of the transfected cells avoiding the generation of a GFP fusion protein that could lead to artefactual localizations (Figure 1A, 1B and Figure S1). A recent study reveals that RNA transcripts with expanded CAG repeats can be translated in the complete absence of a starting ATG [33]. Thus, we evaluated whether the constructs lacking translation initiation codon expressed polyglutamine, using the anti-glutamine monoclonal antibody 1C2 (Figure 1B). The different HTT-e1 constructs were efficiently expressed, as shown by PCR amplification of HTT-GFP (Figure 1B left panel). However, we only detected a polyglutamine track in the constructs containing the ATG starting codon, suggesting that repeat-associated non-ATG translation (RAN translation) is not compatible with the type of vector used to clone the different HTT-e1 forms, at least for polyglutamine production. Since RAN translation can occur in all frames [33], the possibility that CAG expansion produce homopolymeric polyalanine and polyserine proteins cannot be ruled out. It is worth mentioning that 1C2 antibody does not allow quantitative comparison of the levels of 23*CAG-Prot versus 80*CAG-Prot; thus, the differences in the intensity of the 1C2 detected bands is a consequence of the number of glutamines in each HTT-e1, expressed vector (Figure 1B right panel).

We transiently transfected these four different HTT-e1 expressing vectors in differentiated human neuroblastoma cells (SH-SY5Y) as a post-mitotic neuronal cell model. Transfection experiments revealed that CAG expansion in *HTT* mRNA was sufficient to induce a dramatic cytotoxic response in differentiated SH-SY5Y cells (Figure 1C). Cell toxicity assays demonstrated that both CAG-expanded constructs (translated and non-translated forms) drastically affected neuronal cell viability, only differing in the timing of the response, that was earlier for the 80*CAG-RNA construct. However, a expanded polyglutamine expressing vector using CAA instead of CAG repeats (80*CAA), induced a mild toxic effect at the latter time-point that did not reach statistical significance (Figure 1C). These results suggest that the toxic effect induced by the expanded polyglutamine tract is specific for expanded CAG. The *HTT* RNA toxicity was further confirmed with the analysis of early and late apoptotic markers. The results obtained revealed that the expression of CAG-expanded HTT-e1 RNA is sufficient to induce nuclear condensation (Figure 1D) and caspase 9 activation (Figure 1E), processes previously reported to occur in HD brain samples [34], [35]. On the contrary, 80*CAA expressing vector induced milder caspase 9 activation. These data point to a direct link between the toxic effect of expanded *HTT* RNA and an intrinsic apoptotic process.

### Expanded HTT generates small CAG-repeated RNAs with cytotoxic activity

**Figure 2:**
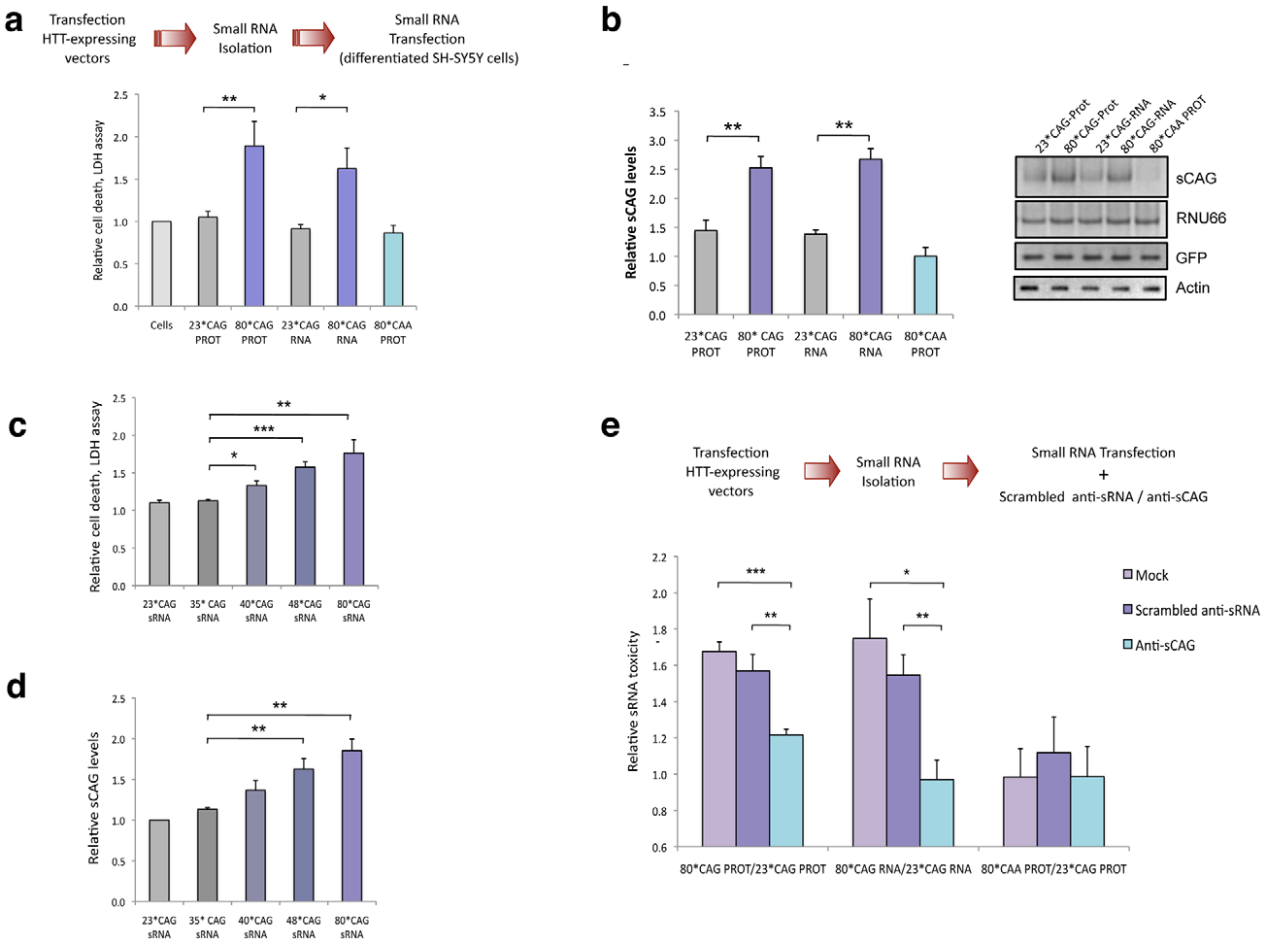
Expanded HTT generates CAG-repeated sRNAs with toxic activity. A. sRNA fraction (<100 nt) were isolated from cells expressing HTT-e1 constructs and equal amounts of each pool were transfected. Both 80*CAG-RNA and 80*CAG-PROT- derived sRNA pools induced death of differentiated SH-SY5Y cells (n = 5; *p<0.05,**p<0.01). 80*CAA-PROT-derived sRNA pools didn't affect SH-SY5Y cell viability. B. The expression of CAG-expanded HTT leads to an increase in CAG-repeated sRNAs of ∼21-nt (sCAG). sCAG levels were quantified using RNU66 as the reference sRNA, and normalized with respect to GFP expression, which indicates the percentage on transfected living cells 24 hours after transfection (n = 4; **p<0.01). C. HTT sRNA toxicity correlates with the length of the CAG expansion, distinguishing pathogenic and non-pathogenic number of CAG repeats (n = 4; * p<0.05, **p<0.01 ***p<0.001. D. HTT sRNA toxicity correlates with the generation of sCAG species (n = 4; **p<0.01). E. Anti-(CAG)_7_ sRNA (anti-sCAG) prevents cell damage caused by mutant-*HTT*-derived sRNA pool. Control sRNA inhibitors did not mitigate sRNA HTT toxicity (n = 4; *p<0.05, **p<0.01, **p<0.001, determinations were performed in quintuplicates). Values represent mean of the ratio expanded-HTT sRNA toxicity vs non-expanded-HTT sRNA toxicity ± SEM. In A. B. C. and D. values represent the mean fold change with respect to the control non-transfected cells ± SEM and are referred to the control cells lacking HTT expression. In all experiments, cells were processed 24 hours after transfection in all the experiments.

Transcripts containing long hairpin structures composed of CNG repeats are Dicer targets [22], [23]. The resultant sRNA products may trigger aberrant gene silencing with putative downstream detrimental effects. To test whether mutant HTT-e1 toxicity was associated to sRNA related mechanisms, we isolated the sRNA fraction (<100 nt) from cells expressing the 23*CAG and 80*CAG forms of HTT-e1, and transfected these sRNA into differentiated human SH-SY5Y neuroblastoma cells. Cell viability assays demonstrated that the sRNA population obtained from cells expressing 80*CAG-PROT and 80*CAG-RNA constructs, induced a remarkable cell death response (Figure 2A), compared to the sRNA population originated from cells expressing the 23*CAG control constructs. These results indicate that the expression of expanded HTT-e1 RNA is sufficient to deregulate the sRNA profile, thereby impairing neuronal viability. Furthermore, the sRNA fraction of cells expressing 80*CAA-PROT failed to induce cell toxicity, suggesting that the sRNA detrimental effect is linked to expanded constructs containing CAG repeats.

In agreement with previous studies demonstrating the generation of trinucleotide-repeated sRNA from triplet-expanded transcripts [19], [23], the expression of CAG-expanded HTT RNA led to the generation of CAG-repeated sRNAs (sCAG), of around 21 nt long (Figure 2B). The identity of these products was further confirmed by direct sequencing of the PCR products (Figure S2) and northern blotting (Figure S3). However, cells expressing the CAA expanded construct failed to produce sCAG, suggesting that the production of these species is not an experimental epiphenomenon.

Variable penetrance for alleles carrying 36–39 CAG repeats has been noted, but the disease appears fully penetrant when the repeat numbers are above 40 [5]. To confirm the sRNA toxicity in HTT carrying a moderate number or repeats, we generated HTT constructs with 35, 40 and 48 CAG repeats (Figure S4). We performed transfection experiments using the sRNA fractions of cells expressing HTT vectors with 23*CAG (normal), 35*CAG (normal), 40*CAG (pathogenic) 48*CAG (pathogenic) and 80*CAG (model for juvenile HD) and subsequently determined cell viability. The sRNA fraction isolated from 40*CAG, 48*CAG and 80*CAG expressing cells induced a significant toxic effect (Figure 2C). Furthermore, the severity of the toxic effect in differentiated SH-SY5Y driven by the sRNA fractions was associated to the length of the CAG stretch, as previously described for the full protein [36] (Figure 2C). In addition, the pools of sRNAs isolated from 40*CAG, 48*CAG and 80*CAG expressing cells contained progressively increasing amounts of sCAGs when compared with that of the 23*CAG and 35*CAG expressing cells (Figure 2D). These results suggest that sRNAs derived from moderately expanded HTT are sufficient to induce a detrimental response and further indicate that expansions above 40*CAG repeats are enough to produce significantly increased amounts of sCAG and a parallel toxic effect.

To analyse the role of sCAG products in HTT sRNA toxicity, we then co-transfected sRNAs pools derived from cells expressing 23*CAG or 80*CAG vectors along with either antisense RNA oligonucleotides that specifically block the action of sCAG (anti-sCAG), or scrambled inhibitors as negative controls (Scrambled sRNA inhibitors). The toxic effect of 80*CAG- versus 23*CAG-derived small RNA was not affected in cells transfected with a scramble siRNA. However anti-sCAG significantly decreased the detrimental effect of the HTT-e1 expanded constructs (Figure 2E). We therefore propose that the generation of sCAG is a key element in the toxicity mediated by CAG-expanded HTT-e1.

### sCAG levels are increased in HD brain samples

**Figure 3:**
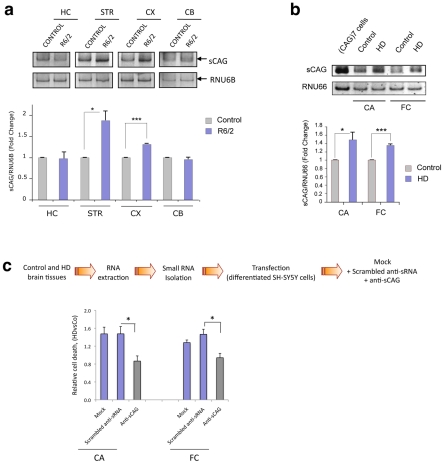
Cytotoxic sCAGs are increased in brain regions of HD. A. sCAG levels are increased in affected brain areas from R6/2 HD mouse model compared to control mice. sCAG were quantified by qRT-PCR using RNU6B as the reference sRNA; HC, hippocampus; STR, striatum cortex; CX, cortex; and CB, cerebellum. Values represent mean fold change with respect the control samples ± SEM (n = 3; *p<0.05 ***p<0.001). B. Increased expression of sCAG in HD human brain samples compared to control subjects. CA, caudate; and FC, frontal cortex. RNU66 sRNA was used as reference sRNA. Values represent mean fold change with respect to the control samples ± SEM (n = 3; *p<0.05 ***p<0.001). C. HD-derived sRNA pools induce neuronal toxicity. sRNA pools were isolated from control and HD human brain samples and delivered to differentiated SH-SY5Y cells; cell death was determined 24 hours later. The use of anti-sCAG dramatically reduced the cytotoxic effect. Control sRNA inhibitors (scrambled anti-sRNA) were used as a negative control. Values represent mean of the ratio (HD sRNA toxicity/Control sRNA toxicity) for each condition ± SEM (experiments were performed in quintuplicates, n = 6; *p<0.05). Pools from four control individuals and four patients with HD were used.

We next examined whether sCAG were detected in different brain areas of the R6/2 HD mouse model, a transgenic line that over-expresses the exon 1 of human HTT with more than 100 repeats, and recapitulates many of the key features found in patients with HD [37]. R6/2 mice of 8 weeks of age exhibited deficits in coordination and activity, striatal atrophy, HTT-aggregate accumulation and down-regulation of striatal-neuron integrity markers [38]. RT-PCR analysis revealed increased sCAG levels in the cortex and striatum of R6/2 mice with respect to their wild-type littermates (Figure 3A), two brain areas preferentially affected in HD. However, no differences in the expression of sCAG species were detected in the cerebellum and hippocampus of R6/2 mice. These results suggest the existence of region specific mechanisms modulating sCAG biogenesis and/or stability in the R6/2 mouse model.

sCAG levels were subsequently analysed in post-mortem brain samples from HD patients and control subjects. RT-PCR analyses confirmed an increase of sCAG in the frontal cortex and caudate regions from HD samples (Figure 3B and Figure S5). The PCR products were sequenced, and sCAG species of 18 nt length were found in both control and HD brain samples. However, sCAG species of 21 nt-long were only detected in HD human brain samples (Figure S6).

To further validate the pathogenic role of sCAG in human brain samples, we isolated the sRNA fractions from control and HD frontal cortex and caudate, and transfected them into differentiated SH-SY5Y cells. HD-sRNAs reproduced the toxicity exerted by the expanded *HTT-e1* sRNAs (Figure 3C). Furthermore, anti-sCAG dramatically diminished the toxic effect of HD-derived sRNA, supporting a pathogenic role of sCAG species produced in HD brains.

### sCAG generation and activity depends on RNAi machinery

In CAG-repeat expansion diseases, Dicer-dependent mechanisms result in the formation of sCAG with putative functions in pathogenic gene silencing [19]. We therefore investigated whether RNAi machinery is involved in the generation and function of sCAG in HD.

**Figure 4:**
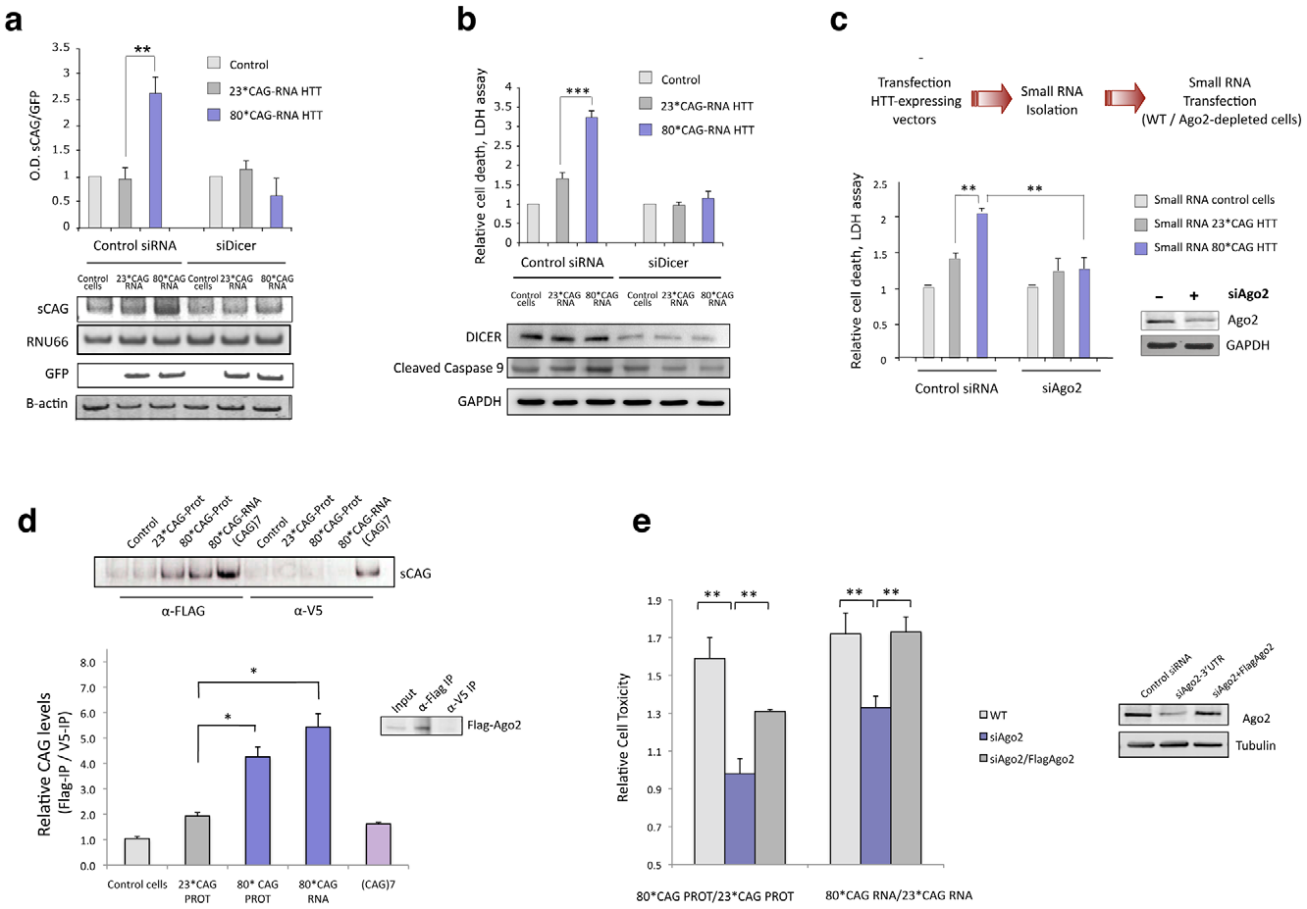
sCAG neurotoxic effect is dependent on Dicer and Ago proteins. A. Dicer knockdown inhibits the generation of sCAGs produced by the expression of 80*CAG HTT-e1. sCAG levels were normalized to RNU66 levels. GFP blots indicate the expression of the HTT-constructs (n = 3; interaction p-value = 0.000138; F = 46.220). B. In the same experiments, cell viability and caspase 9 cleavage analysis show that Dicer depletion mitigates cell death induced by expanded *HTT* (n = 5; interaction p-value = 0.000135; F = 18.263). C. Ago2 depletion mitigates the toxicity of sRNA obtained from mutant *HTT* expressing cells (n = 3; interaction p-value = 0.011; F = 10.821). D. sCAG efficiently associate to Ago2 *in vivo*. *HTT*-expressing constructs were transfected on cells stably expressing Flag-Ago2. Flag IP demonstrate that sCAG binds to Ago2 complex. No significant binding was detected in control IP experiments (α-V5). The plot shows the mean ratio of sCAG levels in FLAG IP *vs.* control V5 IP (n = 3; *p<0.05). E. The expression of Flag-Ago2 in cells depleted for endogenous Ago2 partially, but significantly, rescued CAG-expanded HTT toxic effect (n = 3; *p<0,05). Values represent the mean of the ratio expanded-HTT sRNA toxicity vs non-expanded-HTT sRNA toxicity ± SEM in each experimental condition. Toxicity levels are referred to the control cells lacking HTT expression. In A. B. and C., values represent the mean fold change with respect to the control, non-transfected cells ± SEM. Cells were processed 24 hours after double transfection in all the experiments.

To that end we performed Dicer knockdown experiments in differentiated SH-SY5Y cells that were subsequently transfected with HTTe1-expressing vectors. Dicer depletion prevented the generation of sCAG (Figure 4A and Figure S7) and efficiently mitigated 80*CAG RNA toxicity in SH-SY5Y cells, as indicated by the decrease in LDH release and the inhibition of caspase 9 cleavage (Figure 4B). This result suggest that the toxic effect of the 80*CAG-derived sRNA is caused by a major pathogenic pathway triggered by sCAG.

Since the generation of sCAG was largely dependent on Dicer, we determined Dicer levels in several brain areas of control and R6/2 mice (Figure S8). Dicer expression was significantly decreased in the hippocampus and cerebellum of R6/2 mice while no differences in sCAG levels were detected in these areas (Figure 3A), suggesting that this could be a factor modulating sCAG generation from mutant HTT.

To explore the potential mechanisms of HTT sRNA toxicity and sCAG deleterious effect, we next examined the relationship between 80*CAG toxicity and Ago2 activity, a key factor in miRNA/siRNA gene silencing [39], [40]. Cell viability assays revealed that the toxic effect of sRNA pools originated from cells expressing 80*CAG-PROT and 80*CAG-RNA was diminished in cells depleted of Ago2 (Figure 4C). This result indicates that Ago2 is an important player in the pathogenic effect of 80*CAG-derived sRNA species.

The initiation of a sCAG-mediated gene silencing process requires the incorporation of sCAG into RISC. To test whether sCAG could be loaded into the Ago2 silencing complex, we transfected the HTT-expressing constructs into SH-SY5Y cells stably expressing Flag-Ago2. We performed immunoprecipitation (IP) assays using anti-Flag antibodies for Ago2 IP or anti-V5 antibodies as negative control (Figure S9) and RNA bounded to immunoprecipitated Flag-Ago2 was isolated. The analysis of the Ago2-associated sRNA revealed that sCAG generated from mutant HTT RNA efficiently bound to the Ago2 complex (Figure 4D).

These results, along with the protective role of anti-sCAG, suggest that sCAG initiate a transcriptome-dependent detrimental response through Ago2-mediated gene silencing mechanisms. To evaluate the direct role of Ago2 in the toxic effect of expanded HTT-e1 we restored Ago2 levels in cells depleted of Ago2, and determined cell death (Figure 4E). Restoration of Ago2 levels by the co-transfection of a Flag-Ago2 expressing vector significantly re-established HTT toxicity (Figure 4E).

In humans, the Ago subfamily consists of Ago1, Ago2, Ago3 and Ago4 that guide both siRNAs and miRNAs to complementary sites on target RNAs to modulate their expression [41]. We therefore asked whether Ago2 was the critical mediator on HTT sRNA toxicity or other Ago proteins could be participating as well. Given that Ago3 and Ago4 are not significantly expressed in SH-SY5Y cells (data not shown), we analyzed Ago1 contribution in HTT toxicity. The toxic effect of expanded HTT-e1 was significantly decreased in cells with reduced levels of Ago1, suggesting that mutant HTT effect is also mediated by Ago1 (Figure S10). Since Ago2 is the only member of the Ago family with endonucleolytic activity [40], [42], the results linking both Ago2 and Ago1 with HTT toxicity suggest that sCAG may be modulating gene expression through target mRNA degradation and/or translational inhibition, as described for miRNAs [43].

### sCAGs induce neurotoxicity

**Figure 5:**
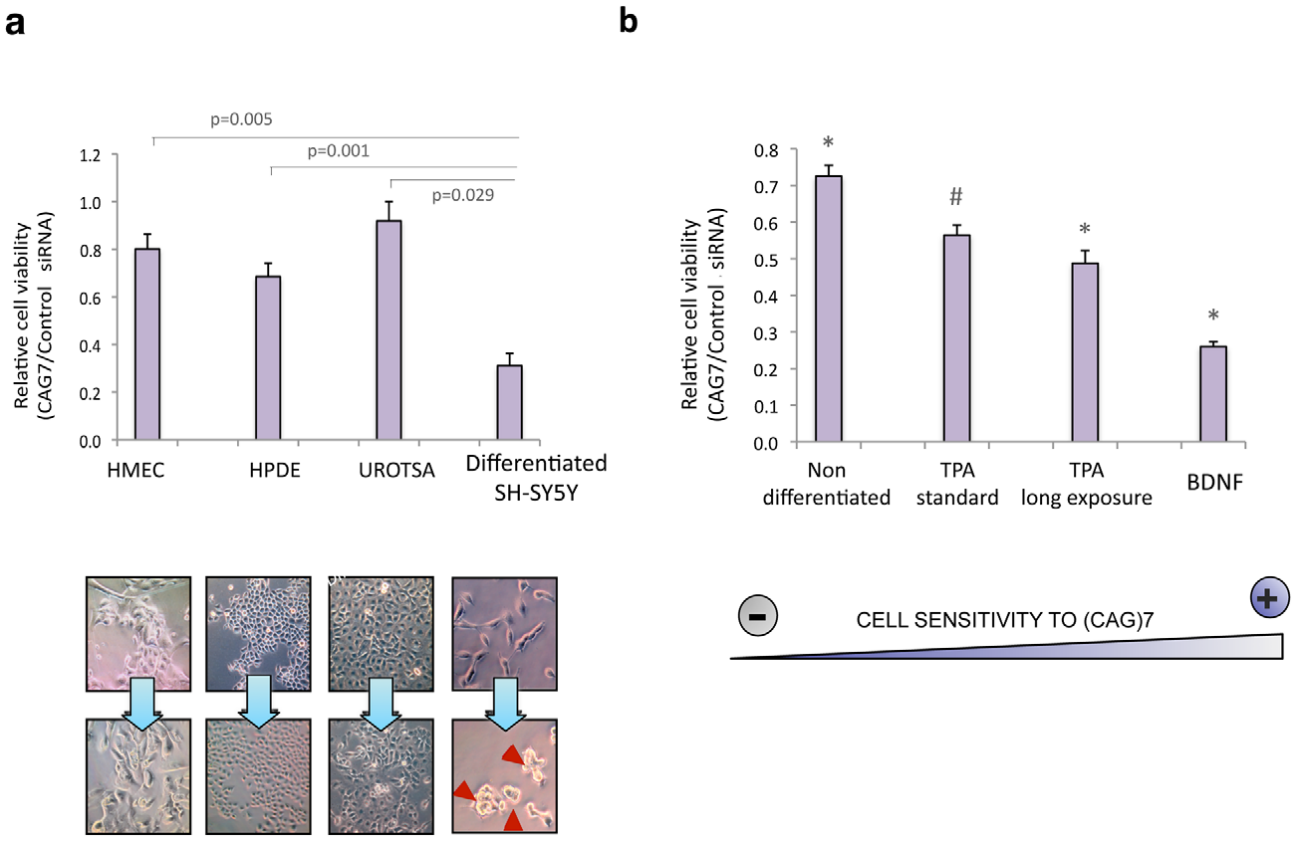
sCAG toxicity is variable in different human cells, preferentially affecting neuronal viability. A. Exogenous administration of (CAG)_7_ siRNAs interfere with cell viability depending on the cell type (n = 3, p<0.0083). HMEC, HPDE and UROTSA cell lines were used as a source for breast, pancreatic and bladder primary human cells. Differentiated SH-SY5Y cells were used as a post-mitotic neuronal cell model (n = 3; one-way ANOVA *p<0.05 ***p<0.001; F = 15.203). B. The toxic effect of (CAG)7 siRNA is dependent on the type of differentiation. SHSY5Y cells were subjected to several neuronal differentiation protocols, and CAG)_7_ or scrambled sequences (control siRNA) were administered in each situation. SH-SY5Y sensitivity to (CAG)7 significantly differs in each differentiation condition (*), excepting for TPA standard differentiation condition (#), whose effect wasn't significantly different from the effect observed under TPA long exposure conditions (One-way ANOVA; F = 63.926). (n = 3, p<0.0083). MTT assays were performed 48 hours after transfection. Graphs show relative cell survival indicated as the ratio between cell viability in cells transfected with controls siRNA *vs* cell viability in cells transfected with (CAG)7. Values indicate the mean ratio ± SEM of three independent experiments.

To validate the possible detrimental effect of sCAG in human cells, a synthetic 21-nt long CAG-repeated siRNA, [(CAG)_7_ siRNA], was delivered to a panel of primary human cell lines including, breast (HMEC), bladder (UROTSA) and pancreatic cells (HPDE). Differentiated SH-SY5Y cells were used as a neuronal model. (CAG)_7_ siRNA impaired cell viability at variable levels in different cell types. Although these results indicate that (CAG)7 siRNA detrimental effect is not restricted to SH-SY5Y cells, this cell model displayed significant higher sensitivity to (CAG)7 (Figure 5A and Figure S11). We performed additional experiments in SH-SY5Y cells following several differentiation protocols that result in differential cell morphology (Figure 5B and Figure S12). These assays demonstrated a correlation between the type of differentiation of SH-SY5Y cells and the sensitivity to (CAG)_7_ which supports a transcriptome-dependent response in sCAG-mediated toxicity.

### Expanded HTT induces gene silencing of CUG-rich transcripts

To validate the gene silencing activity of sCAG, and determine whether a full or partial complementary with the target genes was needed, we generated firefly luciferase-expressing vectors carrying a (CTG)_14_ stretch in the luciferase 3′UTR. We also developed constructs with the sequence (CAG)_14_, which offer an interrupted binding to sCAG. In an attempt to evaluate the consequences of an imperfect matching, we also cloned the sequences 5′-TCCGTGCTGAGCCTGCCTGTCGTCTGTG-3′ and 5′-TGCTAGTATCAGATCTGCTGTGGAATTG-3′, present in the genes *ADORA2A* and *MEIS2* respectively. These two genes are downregulated in affected brain areas of HD patients and brains from the R6/2 mouse model [44]. Furthermore, *in silico* analysis of the sCAG and MEIS2 or ADORA2 duplex stability using RNA hybrid suggests that MEIS2 and ADORA2 could be putative targets of sCAG.

**Figure 6:**
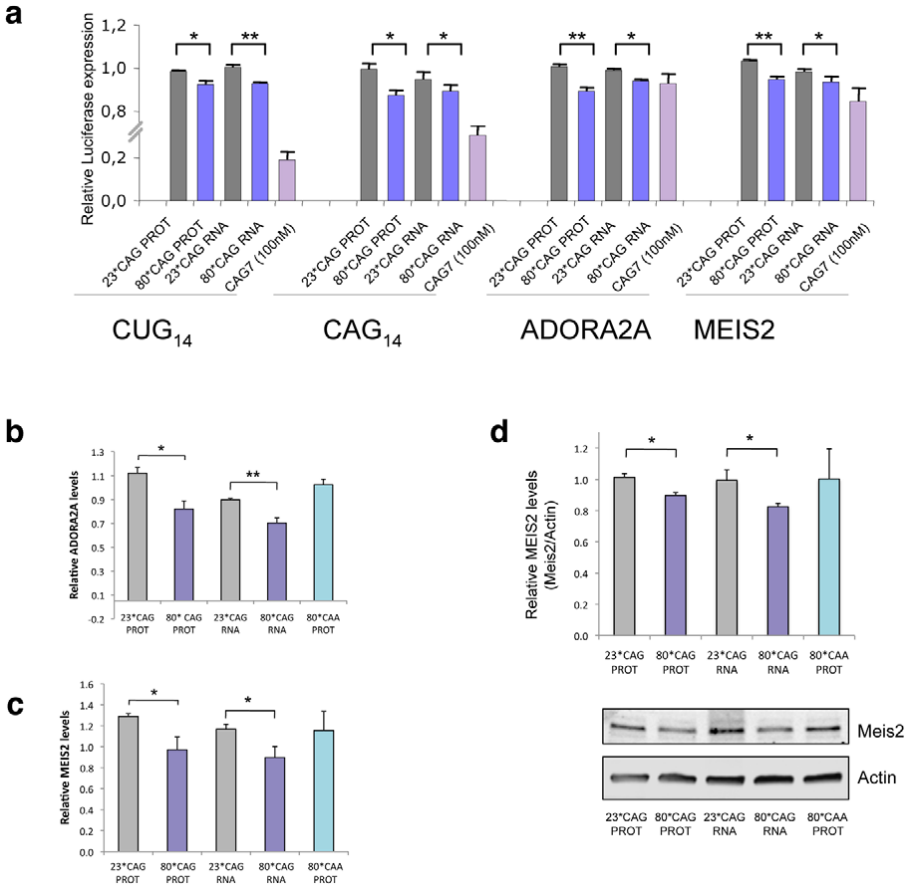
sCAGs induce post-transcriptional gene silencing in genes with CTG regions. A. Hela cells were cotransfected with firefly luciferase expressing vectors containing the indicated nucleotide sequences in its 3′-UTR, the specific HTT-e1 expressing vectors or the (CAG)7 siRNA and Renilla luciferase plasmid to normalize data. Assays were performed 24 hours after transfection. Data were first normalized to the 100% of luminescence obtained with the control luciferase vector, lacking 3′UTR inserts (n = 3; *p<0.05, p**p<0,01). B,C. Levels of ADORA2A and MEIS2 transcripts in SH-SY5Y cells transfected with normal and expanded HTT vectors. MRIP was used as endogenous control. qRT-PCR was performed in cells fixed 24 hours after transfection. (n = 3; *p<0.05). Values represent the mean fold change with respect to the control, non-transfected cells ± SEM. D. Western blot showing reduced MEIS2 protein levels in differentiated SH-SY5Y expressing expanded *HTT* RNA 24 hours after transfection. The graph shows the densitometry determination of MEIS2 levels *vs* β-Actin. Results represent the mean arbitrary optical density change normalized to the mean value obtained in non-transfected cells (n = 4; *p<0.05).

HeLa cells were co-transfected with the different combinations of HTT-expressing vectors and the luciferase vectors, and luminescence was measured 24 hours after transfection. Expanded HTT RNA was able to moderately silence luciferase expression in a construct containing a CTG_14_ sequence in the 3′UTR, compared to control luciferase vectors, and the non-expanded forms of HTT-e1 (Figure 6A). These experiments suggest that sCAGs derived from expanded HTT are involved in post-transcriptional silencing of genes containing CTG repeated tracks. In addition, we also detected a moderate reduction of luciferase activity in constructs harbouring the sequence CAG_14_, suggesting that, expanded-HTT-e1 targets genes with CAG repeats, although the mechanism related may differ from the canonical miRNA/siRNA silencing pathways.

Interestingly, HTT construct expressing 80*CAG moderately decreased the expression (10% of reduction) of the reporters containing *ADORA2A* and *MEIS2* regions (Figure 6A). This result indicates that full complementary between sCAG and its target genes are not needed to induce gene silencing. Therefore, sCAG may behave as siRNA molecules, but also as miRNA-like species, and offer an additional explanation for the broad gene expression deregulation observed in HD [44]. This possibility was further confirmed by RT-PCR quantification of ADORA2 and MEIS2 expression in SH-SY5Y cells transfected with the *HTT*-e1 expressing vectors. (Figure 6B and 6C). The results obtained reproduced the decrease observed in the luciferase assays. Accordingly, the expression of CAA expanded constructs, which failed to generate sCAG, didn't affect *ADORA2A* or *MEIS2* expression levels. We also evaluated if the 80*CAG construct silenced the expression of genes containing a CUG tract, including DMPK, ASTN2 and ZFR (Figure S13). The expanded HTT-e1 induced variable silencing of the different genes that did not correlate with the number of CUG repeats. DMPK is the transcript with higher number of CUG repeats in the 3′UTR with 13 CUG repeats; ASTN2 presents a moderate number of consecutive CUG repeats and ZFR transcripts contains a region harboring 4*CAG immediately followed by 5*CTG. The variability in the dowregulation response suggests that the number of CUG repeats it's not a key factor in mutant HTT-e1 silencing activity.

We evaluated a possible enrichment in CTG regions (of a minimal size of 7) either in the full transcript or in the 3′-UTR of HD downregulated genes. For this analysis we considered the downregulated genes (<−1,2 downregulation and p<0,05), upregulated genes (>1,2 upregulation and p<0,05) and the group of genes that did not show significant expression deregulation, provided in the study by Hodges et al [44]. No significant enrichment in genes containing CTG regions was detected in the downregulated, upregulated or non-regulated genes (X-square p>0,05), suggesting that the overall mRNA gene expression deregulation was dependent on several pathogenic factors besides sCAG-mediated gene silencing.

We next asked whether sCAG could be inducing gene silencing by target mRNA degradation or by translation inhibition. The levels of MEIS2 protein were analyzed in differentiated SH-SY5Y cells transfected with normal and expanded *HTT*-e1. Cells were lysated 24 hours after transfection, a time point in which CAG-expanded HTT RNA toxicity was validated. Given that neural cells are more sensitive to HTT-e1 expression and cell death can be detected 21 h after transfection, MEIS2 levels were normalized by Actin and also referenced to GFP expression, which indicates the percentage of transfected living cells at the time of the analysis. Figure 6D shows MEIS2 protein levels after performing this analysis, confirming that CAG-expanded HTT-e1 induce a reduction in MEIS2 levels by 10%, in agreement with the luciferase reporter assays and mRNA quantification. The decrease in MEIS2 protein levels is similar to the reduction in *MEIS2* mRNA level, which may suggest that mRNA degradation is the main mechanism in the particular case of MEIS2 post-transcriptional gene silencing. However, an exhaustive study should be performed to fully identify sCAG targets and characterize the mechanisms of gene silencing in each particular case.

## Discussion

The latest evidences suggest that RNA detrimental effects contribute to neurodegeneration in a number of trinucleotide repeat expansion diseases. However, these processes have not been extensively addressed in HD, where pathogenesis has been traditionally thought to involve the mutant HTT protein. Our results suggest an RNA pathogenic mechanism in HD that involves the aberrant generation of sCAG RNA species with an inherent toxic effect in a neuronal cell model. We have shown that the generation of sCAG species from expanded HTT exon 1 is largely dependent on Dicer, in agreement with previous studies showing that triplet repeats formed by CNG units adopt hairpin structures that become sliced to sCNG by dicer [22], [23]. In addition, it has become apparent that most of the expanded repeat disease loci have transcription occurring from both strands, raising the possibility that the complementary repeat RNAs form double-stranded structures susceptible to be processed by Dicer. Recently, a natural antisense transcript for *HTT* (*HTTAS*) has been described, covering the exon-1 CAG repeat [45]. Although *HTTAS* is under the control of a weak promoter, it is expressed throughout the brain and other tissues. Therefore, the production of sCAG in HD brains shown in the present study and in fibroblasts of HD patients [22] may originate both from *HTT* expanded hairpin structures and *HTT/HTTAS* double stranded RNAs.

Importantly, CAG repeat lengths above the threshold for complete penetrance (40 or greater) generated increased amounts of sCAG compared with non-pathogenic repeat lengths. Furthermore, our data suggest that the generation of sCAG correlated with the length of the repeat, being sCAG levels progressively higher in cells transfected with HTT-exon-1 constructs harboring 40, 48 and 80 CAG repeats, respectively. This correlated with a gradually increasing detrimental effect driven by the small RNAs fraction of cells expressing HTT-e1 with 40, 48 or 80 CAG repeats, respectively. These results agree with the increased severity of the disease in HD cases presenting extremely long CAG expansions in the *HTT* gene [46].

The amount of sCAG products was not equivalent in different brain areas in a HD mouse model, where increased sCAG levels were detected in the more affected areas. Our data suggest that decreased levels of Dicer could contribute to explain the lack of sCAG increase in the hippocampus and cerebellum of R6/2 mice. However, It is worth mentioning that Dicer activity is subject to regulation that affects the accumulation of miRNAs and probably sCAG. Recent work has identified a battery of proteins that regulate processing either interacting with Dicer or with miRNA precursors, being the activity of some regulators restricted to specific miRNA families [47]. In this context, whether Dicer is particularly active in the cortex and the striatum under basal conditions and/or in HD, the possible mechanisms modulating Dicer activity in specific areas and/or diseased state and its relevance to human disease are open questions that deserve specific research.

Our data indicate that the toxic effect of the sRNA fraction generated by expanded HTT is dependent on Ago proteins and is abolished by anti-sCAG. Furthermore, increased levels of sCAGs were found in Ago2 immunoprecipitates of cells expressing expanded HTT-e1, suggesting that sCAG-driven gene silencing may underlie *HTT*-RNA toxicity. In agreement with RISC-dependent mechanisms, expanded HTT-e1 constructs moderately silenced genes showing pure and interrupted CUG tracks, complementary to sCAG. However, we did not detect a significant enrichment in mRNAs harboring CUG-tracks among those found to be downregulated in human HD brain samples [44]. This suggests that gene expression perturbation in HD brains may reflect primary and secondary pathogenic triggers. In addition, the possibility that sCAG may act through translational repression, a gene silencing mechanism also described for miRNAs [43], cannot be ruled out.

Interestingly, expanded HTT induced similar silencing when using CAG repeats as the target sequence in the luciferase assay. The main structural requirements for gene targeting in miRNA-RISC mediated gene expression regulation are well defined for the most expressed miRNAs, including seed region perfect pairing in the 3′-UTR of the target genes [48]. However, knowledge about the determinants governing gene targeting is far from complete. In fact, targeting can occur through sites other than the 3′-UTR and seed region base pairing is not always required [49]. Whether imperfect base pairing between the CAG tracks in the small RNA and the target genes is compatible with the location and configuration of the sCAG-RISC complex, is an interesting question that should be specifically addressed.

In addition, since trinucleotide repeats have been shown to bind proteins, additional functions for sCAGs should be considered. Gene expression modulation by miRNAs recently included a decoy function, where miRNAs bind to proteins that regulate gene expression, thus modulating their activity [50]. The characterization of the sCAG binding proteins that could have consequences in gene expression regulation may shed light to possible additional RNA related pathogenic mechanisms.

**Figure 7:**
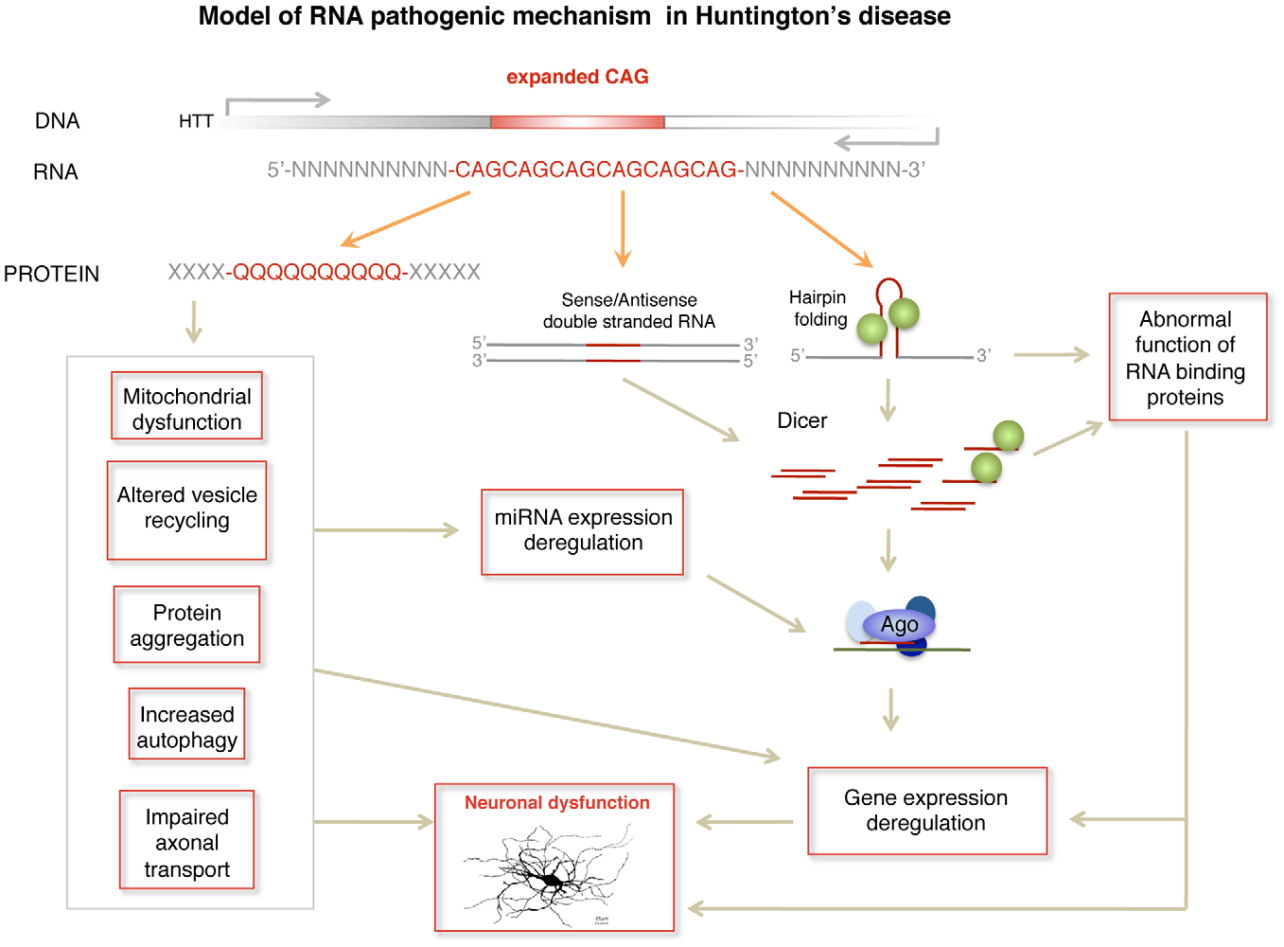
Model of RNA pathogenic mechanism in HD. Several RNA dependent mechanisms contribute to HD pathogenesis. Dicer activity on hairpin-like structures in the mutant HTT gene or in double stranded sense and antisense transcripts induces the formation of sCAG or CAG/CTG siRNA that are incorporated into the RISC complex and trigger abnormal gene silencing. In addition mutant HTT mRNA may induce gene expression deregulation through sequestration of RNA binding proteins that have affinity for CAG repeats, including the transcriptional regulator MBLN. miRNA deregulation produced at least by cellular stress and REST transcriptional malfunction may also contribute to gene expression deregulation in HD.

In summary, we propose a pathogenic RNA dependent mechanism in HD by which sCAG produced over a threshold are neurotoxic. In HD, this mechanism may complement other RNA dependent processes including miRNA deregulation [28]–[32] and possible alterations in alternative splicing driven by MBNL1 sequestration [26], [51] (Figure 7). The detrimental effect may depend not only on the amounts of sCAG generated, but also on the target transcriptome and factors modulating RISC function. These aspects may contribute to sCAG variable vulnerability in different human cells observed in the present study. sCAG induced pathogenesis may underlie common phenotypes in triplet repeat diseases showing CAG expansions in different coding RNAs (leading to polyglutamine expansions in several proteins) and untranslated RNAs [19]. The identification of the specific sCAG-targeted genes and the cellular processes affected by sCAG should pave the way for the development of new therapeutic approaches for HD and other CAG-repeat expansion diseases.

## Materials and Methods

### Cell culture

Human Mammary Epithelial Cells (HMEC) were maintained in MEBM medium supplemented with Bullet-kit (Lonza), Human Pancreatic Duct Epithelial Cells (HPDE) were cultured in KSFM medium (Invitrogen) supplemented with epithelial growth factor (0.1–0.2 ng/mL) and bovine pituitary extract (25 µg/mL). UROTSA cells were maintained in RPMI medium (Invitrogen) supplemented with 10% FBS (Fetal Bovine Serum, Invitrogen). HeLa cells and SH-SY5Y neuroblastoma cells were maintained in Dulbecco's Modified Eagle's Medium (DMEM, Invitrogen) supplemented with 10% FBS, 2 mM L-glutamine, 100 units/ml penicillin and 100 µg/ml Streptomycin (GIBCO, Invitrogen). In the case of SH-SY5Y cells, FBS was heat inactivated for 45 min at 56°C prior to use. Unless otherwise indicated, SH-SY5Y cells differentiation was performed culturing the cells in the standard growing medium containing 10 µM retinoic acid (RA) during four days. The media was then replaced by fresh medium containing 80 nM of 12-O-tetradecanoylphorbol-13-acetate (TPA) during five additional days [52] Different neuronal differentiation protocols are provided in Figure S8).

### Generation of HTT-e1–expressing vectors

Different forms of the exon 1 of the *HTT* gene (HTT-e1) differing in the CAG repeat length (23*CAG-, 35*CAG-, 40*CAG-, 80*CAG- or 80*CAA-PROT; and 23*CAG-, 80*CAG-RNA) were synthesized by Geneart. Flanking *EcoRI* restriction sites were added during the synthesis that were used to sub-clone the HTT-e1 variants into the pIRES2-EGFP vector (BD Biosciences, Clontech). Not-translatable constructs lack the translation initiation codon (AUG) and the second methionine (AUG) found in HTT exon1 (Figure 1A).

### Transfections

All the transfection experiments were performed using Lipofectamine 2000 (Invitrogen), according to the manufacturer's instruction and at a 60% cell confluence. (CAG)_7_ (5′CAGCAGCAGCAGCAGCAGCAG-3′) and control, scrambled siRNA (5′-GCGACGUUCCUGAAACCAC-3′) were purchased from Dharmacon and were administered at a final concentration of 50 nM, unless otherwise indicated. The anti-sCAG small RNA (LNA modified 5′-(CTG)_7_ ), and scrambled sequences (LNA modified 5′-GTGTAACACGTCTATACGCCCA-3′) were ordered from Exiqon. Both anti-sCAG and the corresponding scrambled inhibitor were transfected at a final concentration of 60 nM. Transfections with sRNA pools were performed using 35 ng of each sRNA pool per well (quintuplicates, 96wells multiwell). Dicer, Ago1 and Ago2 knockdown experiments were performed by a double transfection procedure; consisting in the transfection of the Scrambled, Ago2 or Dicer siRNA in the first assay (50 nM), and the co-transfection of the siRNA and HTT construct 48 hours later at 75 nM and 400 ng, respectively, in MW6 plates. Dicer siRNA (5′- GCUCGAAAUCUUACGCAAAUA-3′), Ago1 siRNA (5′- CAUCAGGACUGUUGAGUAA -3′) and Ago2 siRNA (5′-GCACGGAAGUCCAUCUGAA-3′) were purchased from Dharmacon. A siRNA against the 3′UTR of Ago2 (siAgo2-3′UTR: 5′-GGAAATATGGTTTGCTAAA-3′) was used in the HTT toxicity rescue experiments (Figure 4E). Transfection efficiency in experiments using siRNA or sRNA pools was determined at each experimental condition using siGLO transfection indicator (Dharmacon). Transfection conditions were optimized in order to obtain similar transfection efficiencies (∼90%) in all the cell lines analyzed.

### FLAG-Ago2 stable cell lines

SH-SY5Y cells were transfected with a Flag/HA-AGO2 expressing vector (Flag-tagged Ago2 expression vector was kindly provided by Prof. R. Shiekhattar). The plasmid encodes for a neo-resistance marker and transfected cells were grown in the presence of 800 µg/ml of Geneticin (G418, *Gibco* Laboratories) for 10–14 days. Single clones were selected to generate monoclonal cell lines. Expression of Flag/HA-AGO2 protein was checked by western blot and immunofluorescence in several cell clones.

### Western blotting

For protein extraction, cells were rapidly rinsed with ice-cold PBS and solubilized with a lysis buffer described in [53]. Cells were then scraped off, incubated on ice for 15 min and centrifuged at 14000 rpm for 15 min. Samples were resolved in 10% SDS-PAGE gels and transferred to nitrocellulose membranes using the iBlot Dry Blotting System (Invitrogen). Membranes were blocked for 1 h with 10% skimmed milk in TBS (Tris-HCl, pH 7.5, 10 mm; NaCl, 100 mm) containing 0.1% Tween-20 (TBS-T). Membranes were incubated at 4°C and overnight with primary antibodies (diluted in TBS-T). After washing with TBS-T, membranes were incubated for 45 min at room temperature with the appropriate secondary antibodies (diluted in TBS-T), and then washed again with TBS-T. Detection was performed by ECL Western blotting detection reagent (Amersham Bioscience). Chemiluminescence was determined with a LAS-3000 image analyzer (Fuji PhotoFilm Co., Carrollton, TX, USA). Primary antibodies were anti-polyQ (MAB1574, 1∶1000, Millipore), anti-GFP (1∶2000, Molecular Probes, rabbit), anti-PARP (1∶5000, BD Pharmigen, mouse), anti-cleaved caspase 9 (1∶1000, Cell Signaling, rabbit), anti-Dicer (1∶500, Abcam, mouse), anti-Ago2, (1∶500, Abnova, clone 2E12-1C9). Anti-Ago1 antibody (1∶1000, rat) was kindly provided by Dr. G. Meister . Anti-GAPDH (1∶4000, Chemicon, mouse), anti-α-Actin (1∶5000, Chemicon, mouse) and anti-α-Tubulin (1∶50000, Sigma, mouse) were used as loading controls. Secondary antibodies were HRP-conjugated anti-mouse, anti-rat and anti-rabbit (1∶2000, DAKO)

### Immunofluorescence

SH-SY5Y cells grown on coverslides were rinsed several times with PBS and fixed for 20 min at room temperature with 4% paraformaldehyde in PBS. After rinsing, cells were permeabilized for 20 min in 0.5% Triton-X-100 in PBS. Non-specific binding sites were then blocked by incubating for 1 h in PBS containing 0.2% Triton X-100 and 10% FBS. Incubation with primary mouse anti-PolyQ antibody (1∶2000, Millipore, clone 5TF1-1C21) was carried out overnight at 4°C in PBS containing 0.2% Triton-X-100 and 1% FBS. After washing, coverslides were incubated with secondary anti-mouse IgG Alexa 555 IgG (Molecular Probes) at a dilution of 1∶1000 for 1 h at room temperature. After washing, coverslides were mounted in Vectashield-DAPI solution, and cells visualized under a Leica microscope (DMR). Images were captured using a digital camera (Leica DC500).

### Cell viability assay

Differentiated SH-SY5Y cells were transfected in 96 well plates and cell viability was determined 24 hours post-transfection with the 3-(4,5- dimethythiazol-. 2-yl)-2,5-diphenyl tetrazolium bromide (MTT) assay. MTT was added to cell culture media at 0.5 mg/mL final concentration and incubated for 40 minutes at 37°C. Cells were then lysed with 100 µL of DMSO upon medium removal and absorbance was measured at 550 nm. In each experiment, determinations were performed in tetraplicates.

### Cell toxicity assays

Lactose dehydrogenase (LDH) released from dying cells was determined using the LDH assay (Cytotox 96, Promega) according to the manufacturer's protocol, at different time-points following transfection (see figure legends). Absorbance was recorded at 490 nm. LDH determinations were performed in quintuplicate.

Cell death was also determined with the simultaneous staining of alive and dead cells using fluorescein diacetate (FDA) and propidium iodide (PI), respectively in a double staining procedure. Cells were rinsed with PBS 1× and then incubated for 45 s at 22–25°C with 15 mg/ml FDA (Sigma) and 4.6 mg/ml PI (Molecular Probes, Inc., Eugene, OR, USA) in PBS. The staining solution was replaced by PBS 1× and the stained cells were immediately examined under a Leica microscope.

### Immunoprecipitations assays and sRNA extraction

Ago2 Immunoprecipitations assays and the extractions of the Ago2-bounded RNA were carried out as described previously [54]. Flag-Ago2 immunoprecipitation was performed using ANTI-FLAG M2 affinity gel (Sigma). ANTI-V5 affinity gel (Sigma) was used as a negative control for Ago2 IP. sCAG levels were determined by polyadenylation and RT-PCR. SH-SY5Y cells transfected with 100 nM (CAG)_7_ were used as a positive control.

### RNA isolation

Total RNA from cells or brain tissues was extracted using miRNeasy Mini kit (Qiagen). Small RNA species (<100 nt), were fractionated by size-exclusion column chromatography using Microcon Y-10 (Millipore) according to the manufacturer's instructions.

### RNA polyadenylation

Total RNA was treated with TURBO DNA-free kit (Ambion). *In vitro* polyadenylation reactions were carried out using 1 µg of total RNA or 100 ng of sRNA enriched fraction and poly(A) polymerase (Ambion) for 1 h at 37°C in the presence of ATP (1 mM). Samples were then annealed with a polyT-adapter primer (5′-CGAATTCTAGAGCTCGAGGCAGGCGACATGGCTfGGCTAGTTAAGCTTGGTACCGAGCTCGGATCCACTAGTCCTTTTTTTTTTTTTTTTTTTTTTTTTAC-3′) prior to RT reaction. Specific primers recognizing the adapter and sCAG allowed the amplification of specific products by RT-PCR.

### qRT–PCR

sCAG expression levels in cells transfected with the non-expanded or expanded *HTT-e1* were analyzed by RT-PCR or densitometry of the PCR amplified products. Total RNA, polyadenylated total RNA or sRNA was retrotranscribed using the Superscript III RT kit (Invitrogen).

Equal amounts of cDNA were mixed with SYBR Green PCR mix (Roche). Five pmol of the forward primer (designed on the CAG repeat sequence) and reverse primer (based on the adaptor sequence) were used in each reaction. Amplification was done under the conditions of 15 sec at 95°C and followed by 55 cycles consisting in 1 min at 60°C and 2 min at 72°C in a LightCycler 480 Real-Time PCR System (Roche). The entire experiments were repeated three times on independent RNA preparations. RNU66 expression was used as a reference small RNA. Values were also referenced to the GFP levels, which refers to the number of transfected living cells at a particular time. β-Actin was the endogenous reference gene for GFP normalization. Data are presented as the ratio between the normalized expression of sCAG (sCAG/RNU66) or a particular gene (gene/β-Actin) and the normalized expression of GFP (GFP/β-Actin).

sRNA qRT-PCRs were performed with the following set of primers: sCAG Forward: 5′-CAGCAGCAGCAGCAGCAG-3′, sCAG Reverse: complementary to the polyT adapter after polyadenylation (5′-CGAATTCTAGAGCTCGAGGCAGG-3′); RNU66 forward: 5′-GTAACTGTGGTGATGGAAATGTG-3′; RNU66 reverse: 5′- GACTGTACTAGGATAGAAAGAACC-3′; RNU6B forward: 5′-CGCTTCGGCAGCACATATAC-3′; RNU6B reverse: 5′-TTCACGAATTTGCGTGTCAT-3′.

mRNA qRTPCR were performed using the following primer sets: GFP forward: 5′-TGCAGTGCTTCAGCCGCTAC-3′; GFP reverse: 5-TCGCCCTCGAACTTCACCTC-3′; DMPK forward: 5′-TGGGCTACTCCTACTCCTG-3′; DMPK reverse: 5′- AGCTGTTTCATCCTGTGGG-3′; ASTN2 forward: 5′- GACATTCTACACGGAGCAGTAC-3′; ASTN2 reverse: 5′- GTGAGTGGACAAGACATCTGG-3′; ZFR forward: 5′- TGGGACTCAAAATCAGCTACG-3′; ZFR reverse: 5′- TGGTTCTGTTGATGGAATGGG-3′; β-Actin Forward: 5′-CTGGAACGGTGAAGGTGACA-3′; β-Actin Reverse: 5′-GGGAGAGGACTGGGCCATT-3′.

Regular detection of GFP and HTT-e1 expression was performed using the following set of primers GFP Forward/GFP Reverse, and HTT forward//pIRES-GFP reverse (5′-GTCCCTCAAGTCCTTCCAGC-3′/5′-GAACTTCAGGGTCAGCTTCG-3′).

Gene expression analysis of *ADORA2A* and *MEIS2* genes were carried out using Taqman assays (assay ID: Hs00169123_m1* for *ADORA2A* and assay No: Hs00542638_m1* for *MEIS2*). Data were normalized using *MRIP* (assay ID: Hs00819388_m1) as an endogenous reference gene. Amplification was done under the conditions: 15 sec at 95°C and followed by 55 cycles consisting in 1 min at 60°C and 2 min at 72°C on the ABI PRISM 7000 Detection system (Applied Biosystems). The entire experiments were repeated four times on independent RNA preparations.

qPCR results were analyzed using the 2−delta delta Ct method.

### Detection of miRNA precursors and mature forms

The levels of the precursors and mature forms of miR-16 and miR-29 in normal cells and cells with decreased levels of Dicer were determined by polyadenylation and RT-PCR in total RNA, as previously described. The following oligonucleotides were used: for miR-16-1: 5′-TAGCAGCACGTAAATATTGGCG-3′; for miR-29a: 5′- TAGCACCATCTGAAATCGGTT-3′.

### sCAG sequencing

CAG *PCR products* were run on a 15% polyacrylamide gel and visualized by SybrSafe staining (Invitrogene). PCR products were purified and ligated into pGEMT-easy vector. The sequencing reactions of the vectors were carried out using the Big Dye 3.1 Termination Cycle Sequencing Kit and DNA Sequencer (ABI3100) from Applied Biosystems.

### Northern blot

Total RNA (30 µg) or small RNA (<100 nt long, 4 µg) were resolved in a 15% acrylamide-7.5 M urea gel and transferred to Hybond-N^+^ membranes (Amersham Bioscience) in 0.5× Tris-buffered EDTA at 200 mA overnight at 4°C. The membranes were UV cross-linked and heated at 80°C for 1 h. LNA probes (Exiqon) and oligoribonucleotide probes against (CAG)_7_ repeats (5′-CTGCTGCTGCTGCTGCTGCTG-3′) were *labelled* with γ-^32^P-dATP *using Optikinase* (USB Corp.). LNA probe complementary to RNU6B was used as loading control (5′-CACGAATTTGCGTGTCATCCTT-3′, Exiqon) and an oligonucleotide probe complementary to GFP (5′- GAACTTCAGGGTCAGCTTGC) was used to detect the expression of the different pIRES-HTT-e1-GFP vectors (HTT-e1-IRES-GFP transcripts with a length of around 1.5 Kb). Oligonucleotide probes *hybridisation* and washings were *performed* at 50°*C using PerfectHyb Plus buffer (Sigma)*. The membranes were exposed to *Fuji Imaging* plates, scanned with a *FLA*-*5000 PhosphorImager* (*Fuji PhotoFilm Co*.) and quantified with ImageJ software.

### Firefly luciferase assay

*A series of firefly luciferase*-based reporter *constructs* were used for quantitative measurement of sCAG-mediated post-transcriptional gene silencing in genes containing (CUG)_7_/(CAG)_7_ stretches.

The putative target sequences were obtained by the annealing of oligonucleotides with the desired sequence, containing an *XbaI* restriction site at their 5′ end. The resulting double stranded DNA fragments were cloned downstream of the firefly luciferase reporter gene in the pGL4.13 vector (Promega) using XbaI restriction site.

The oligonucleotides used were: 5′-CTAG(CTG)_14_-3′ and reverse 5′-CTAG(CAG)_14_ for genes containing (CUG)_n_ repeats; forward 5′-CTAG(CAG)_14_-3′ and reverse 5′-CTAG(CTG)_14_-3′ for genes containing (CAG)_n_ repeats; forward 5′-CTAGTCCGTGCTGAGCCTGCCTGTCGTCTGTG-3′ and reverse 5′- CTAGCACAGACGACAGGCAGGCTCAGCACGGA-3′ mimicking a CUG rich region located in ADORA2A gene; forward 5′-CTAGTGCTAGTATCAGATCTGCTGTGGAATTG-3′ and reverse 5′-CTAGCAATTCCACAGCAGATCTGATACTAGCA-3′for a CTG containing region of MEIS2 gene .

HeLa cells were seeded at 1.3×10^4^ cells/well in 96-well plates and 24 h later they were co-transfected with the following set of vectors: HTT-e1 constructs (40 ng), Firefly reporter constructs (24 ng) and Renilla reporter plasmid pGL4.75 (3 ng). The pGL4.13 vector without 3′UTR insertion was used as negative control for gene silencing. The (CAG)_7_ mimic was used as a positive control for silencing effect of CUG enriched stretches.

The activity of Firefly and Renilla luciferases was determined 24 h after transfection using the Dual-Glo™ Luciferase Assay System (Promega). Relative reporter activity was obtained by normalization to the Renilla luciferase activity. Each experiment was done in triplicate, and at least three independent experiments were performed for each construct.

### Mice brain samples

Hemizygous male mice transgenic for exon 1 of the human Huntingtin gene with a greatly expanded CAG repeat (R6/2 mice) [37] were purchased from The Jackson Laboratory (Bar Harbor, code B6CBA-Tg(HDexon1)62Gpb/1J; 155–175 CAG repeats). The colony was maintained by back-crossing R6/2 males with (CBA×C57BL/6J) F1 females. Mice were sacrificed at 8 weeks of age, and brain samples were snap-frozen and subsequently stored at −80°C until use. Those 8 week-old R6/2 mice exhibited various hallmarks of HD-like disease, such as motor symptoms (deficits in coordination and activity), neuropathological deficits (striatal atrophy and huntingtin-aggregate accumulation) and molecular-pathology alterations (down-regulation of striatal-neuron integrity markers) [38].

### Human brain samples

Brain samples corresponding to the frontal cortex (FC) and the striatum (dorsal caudate, CA) of HD patients and controls were obtained from the Institute of Neuropathology and the University of Barcelona Brain Bank. CAG expansions ranged from 41 CAG repeats to 62 CAG repeats in the HD samples (control samples harbored less than 23 CAG repeats). The neuropathological examination in HD cases revealed severe atrophy of the caudate and putamen, cerebral cortical atrophy. This was accompanied by marked neuronal loss and astrocytic gliosis. Individual neurons in the cerebral cortex and striatum exhibited ubiquitin-positive intranuclear inclusions typical of diseases with CAG triplet expansions. HD cases were categorized as stage 4 following Vonsattel classification.

### Ethics statement

Animal handling procedures was conducted in accordance with Directive 86/609/EU of the European Commission.

Brain samples of HD patients and controls were obtained from the Institute of Neuropathology and the University of Barcelona Brain Bank, after the informed consent of the patients or their relatives and the approval of the local ethics committee. Ethical issues and legislation as defined by the European Union and national law. All activities were conducted with the approval of responsible ethical committees. The following general guidelines apply:- The Charter of Fundamental Rights of the EU; - Directive 2004/23/EC of the European Parliament and of the Council of 31 March 2004 on setting standards of quality and safety for the donation, procurement, testing, processing, preservation, storage and distribution of human tissues and cells; - Directive 95/46/EC of the European Parliament and of the Council of 24 October 1995 on the protection of individuals with regard to the processing of personal data and on the free movement of such data.

### Statistical methods

In each experiment “n” refers to completely independent experiments. Statistical analyses were performed using the two-tailed unpaired t-student's test for single comparisons (p<0,05) and the analysis of variance (ANOVA) when multiple pair-wise conditions were compared, where ad-hoc tests were addressed with the Bonferroni correction. The ANOVA test included an interaction term in the cases were the aim was to evaluate whether specific proteins modulate HTT-e1 response. Unless specifically indicated, p-values withstand Bonferroni correction.

## Supporting Information

Figure S1 Immunofluorescent detection of HTT expression. SH-SY5Y cells were transfected with the indicated HTT-constructs and expression was evaluated 24 h later. GFP expression (green) allows the identification of HTT-IRES-GFP RNA and protein expressing constructs. Polyglutamine antibody staining (red) labels the cells expressing the HTT protein. Only the 23*CAG-PROT and 80*CAG-PROT constructs express the protein.
(TIF)

Figure S2 (CAG)7 sRNA are detected in cells expressing 80*CAG HTT. sCAG were detected in cells expressing HTT-e1-expressing vectors by RNA polyadenylation, polyT-based RT and PCR (a detailed description is provided in Methods). PCR products were cloned into pGEMT-easy vector and then sequenced. A. Histogram showing the cloned 21 nt-long (CAG)_7_ sRNA. Sequences corresponding to the cloning vector, the polyA region and the RT-PCR adapter oligonucleotide are indicated. B. Table shows the type of sequences identified in the different samples and their frequency. (CAG)_7_ sRNA were only detected in cells expressing 80*CAG HTT (RNA or protein). Sixteen different positive sCAG-cloning colonies were sequenced for each HTT variant.
(TIF)

Figure S3 Increased sCAG levels can be detected by Northern blot. Northern blot analysis confirms the increase in sCAG in cells expressing expanded HTT-e1. A. The expression of the different HTT-e1 expression vectors can be detected by α-CAG and α-GFP probes. RNA18S signal was used as loading control. B. CAG-repeated sRNA of around 21 nt long and longer CAG-rich species are produced in cells expressing expanded HTT-e1 RNA. RNU6B sRNA was used as loading control. Thirty µg of total RNA was used per lane. C. sCAG products were also detected in sRNA fraction obtained from cells expressing expanded HTT-e1 (4 µg of RNA<100 nt were subjected to electrophoresis).
(TIF)

Figure S4 HTT-e1-IRES-GFP vectors are efficiently expressed in SH-SY5Y. Differentiated neuroblastoma cells were transfected with HTT-e1 constructs harbouring 23, 35, 40, 48 or 80 CAG repeats. Western blots shows polyQ and GFP expression. The results are representative of 3 independent experiments.
(TIF)

Figure S5 sCAG levels in individual HD brain samples. Graph shows relative sCAG levels in control and HD human brain samples containing different number of CAG repeats. RNU66 was used as the reference sRNA. RT-PCR assays showed that all the HD brain samples analyzed displayed increased sCAG levels compared to control samples. The results are representative of 3 independent experiments performed in triplicate.
(TIF)

Figure S6 sCAG are detected in HD human frontal cortex. sCAG PCRs from Control and HD frontal cortex were resolved in polyacrylamide gels. Gel fragments corresponding to a size of around 18–24 nt were cut and DNA was isolated and cloned into pGEMT-easy vector. Twelve positive colonies were sequenced for each sample (3 HD-derived brain samples; 3 samples from control subjects). A. Histogram with the sequencing results of HD-derived sample expressing (CAG)_7_ sRNA. B. Table showing the type of sequences identified in the different samples and their frequency. sRNA constituted by 6*CAG repeats were found in both and HD brains. (CAG)7 sRNA were only found in HD-derived samples.
(TIF)

Figure S7 sCAG generation depends on Dicer expression. A. Northern blot confirming that the generation of CAG-repeated sRNAs in cells expressing expanded HTT-e1 is suppressed when cells are depleted of Dicer. RNA6B was used as a loading control. B. Dicing efficiency was determined in normal cells and cells showing low Dicer levels by measuring miRNA maturation. Gels show precursor and mature forms of miR-16 and miR-29a (very abundant and low abundant in SH-SY5Y cells respectively). C. Western blot showing Dicer protein levels in control cells and cells treated with siRNA against Dicer. RNA was isolated 24 hours after transfection with the HTT-e1 expressing vectors.
(TIF)

Figure S8 Dicer levels are significantly decreased in the hippocampus and the cerebellum of R6/2 mice. Protein lysates were obtained from striatum, cortex, hippocampus and cerebellum of control and R6/2 mice (8 weeks old). Dicer protein levels were evaluated in four different control mice and five R6/2. Graphs show the densitometry determination of Dicer levels *vs* Tubulin for each specific brain area. Results demonstrate that Dicer levels are decreased in hippocampus and cerebellum of R6/2 compared to its levels in control mice. Results represent the mean arbitrary optical density change ± SEM (n = 3; *p<0.05).
(TIF)

Figure S9 Experimental design to detect CAG-repeated small RNAs in Ago2 complexes. SH-SY5Y cells stably expressing mild levels of the Flag-Ago2 fusion protein were transfected with HTT-expressing vectors (protein or RNA expressing constructs). Immunoprecipitation (IP) experiments were performed using an anti-Flag tag antibody or an anti-V5 tag antibody as negative control for Ago2 binding. Western blot analysis shows that Ago2 protein was only immunoprecipitated by the use of Flag antibodies. These immunoprecipitates were used for the detection of sCAG (Figure 4D).
(TIF)

Figure S10 Ago1 activity contributes to HTT RNA toxicity. SH-SY5Y cells were transfected with siRNAs specific for Ago1 (siAgo1) or scrambled siRNAs as control. Cells were co-transfected 48 hours later with the combination HTT-e1/siAgo1 or HTT-e1/control siRNA. A. LDH cytotoxicity assay shows that the detrimental effect of CAG-expanded HTT RNA is dramatically reduced when cells are depleted of Ago1 (n = 3; *p<0,05 **p<0,01). Values are referred to the control cells lacking HTT expression. Values represent mean of the ratio: expanded-HTT RNA toxicity vs non-expanded-HTT RNA toxicity ± SEM for each experimental condition.
(TIF)

Figure S11 Cell-type dependent cytotoxic effect of (CAG)_7_ siRNAs. (CAG)_7_ siRNA was transfected into HMEC, UROTSA and HPDE non-neural human cells. Differentiated SH-SY5Y cells were used as a post-mitotic neuronal cell model. (CAG)_7_ siRNA exert a cell-type dependent toxic effect, preferentially affecting neuronal viability. Fluorescein diacetate staining (green) labels living cells. Propidium iodide staining (red) specifically labels dead cells. Stainings were performed 36 hours after transfection (n = 3, each experiment performed in triplicate).
(TIF)

Figure S12 Neuronal differentiation protocols used in SH-SY5Y cells. Schematic description of the different neuronal differentiation protocols used to characterize the toxic effect of (CAG)7 siRNA (Figure 5B). Pictures show the morphological changes induced after each differentiation condition.
(TIF)

Figure S13 Expanded HTT-e1 RNA silences CUG rich genes. Total RNA was isolated from cells expressing the different HTT-e1 expressing vectors 24 hours after transfection. qRT-PCR revealed that expanded HTT-e1 (protein and RNA) can suppressed endogenous levels of CUG rich transcripts (DMPK, ASTN2 and ZFR). The percentage of suppression differs among the transcripts analyzed. (n = 4; *p<0,05, experiments performed in triplicates).
(TIF)
