# Supplementary figures and images for: A Pathogenic Mechanism in Huntington's Disease Involves Small CAG-Repeated RNAs with Neurotoxic Activity
Source: PLoS Genet. 2012 Feb 23;8(2):e1002481. doi: 10.1371/journal.pgen.1002481 (PMC3285580; doi:10.1371/journal.pgen.1002481)

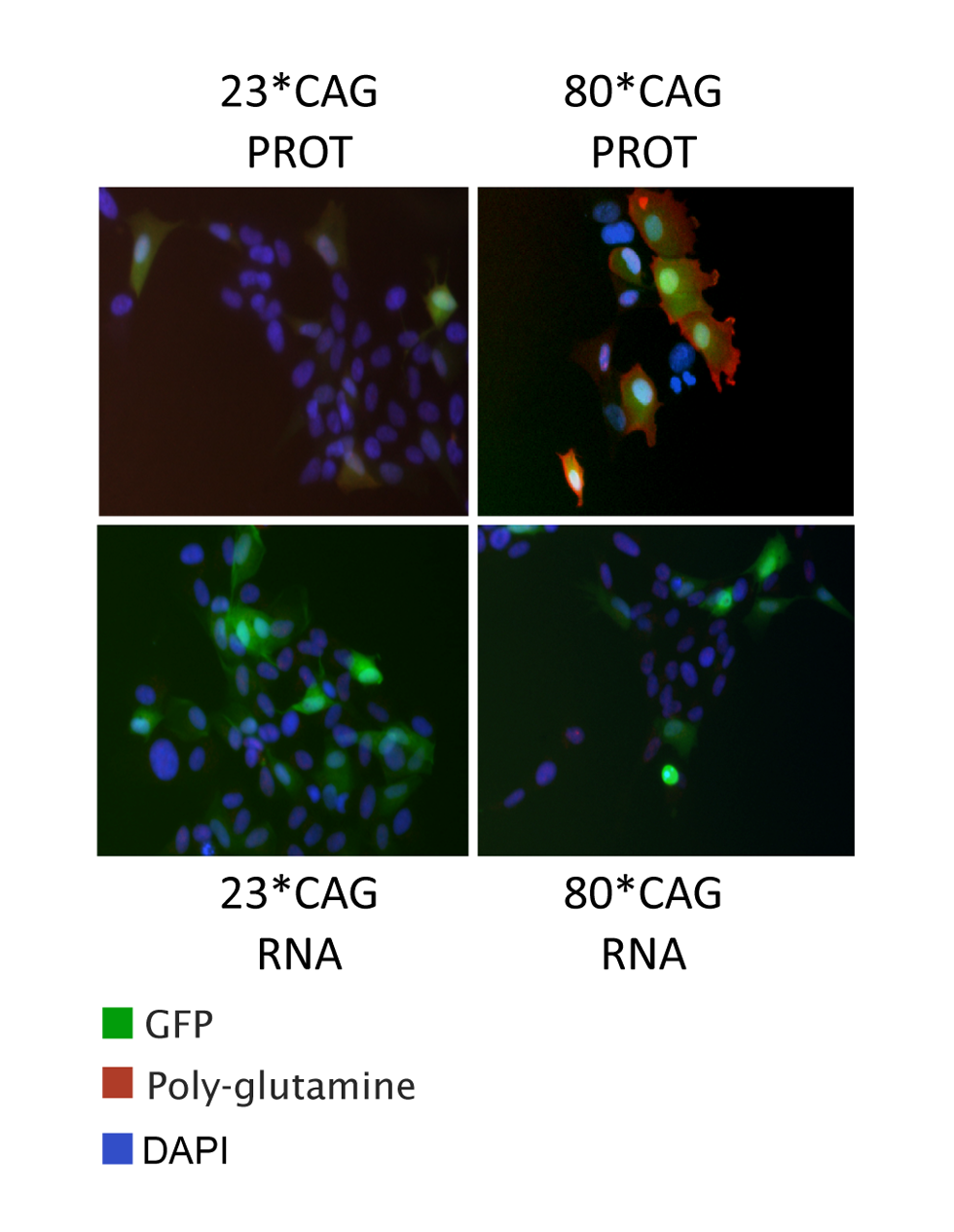

Supplement: Figure S1 — Immunofluorescent detection of HTT expression. SH-SY5Y cells were transfected with the indicated HTT-constructs and expression was evaluated 24 h later. GFP expression (green) allows the identification of HTT-IRES-GFP RNA and protein expressing constructs. Polyglutamine antibody staining (red) labels the cells expressing the HTT protein. Only the 23*CAG-PROT and 80*CAG-PROT constructs express the protein. (TIF) [file pgen.1002481.s001.tif]

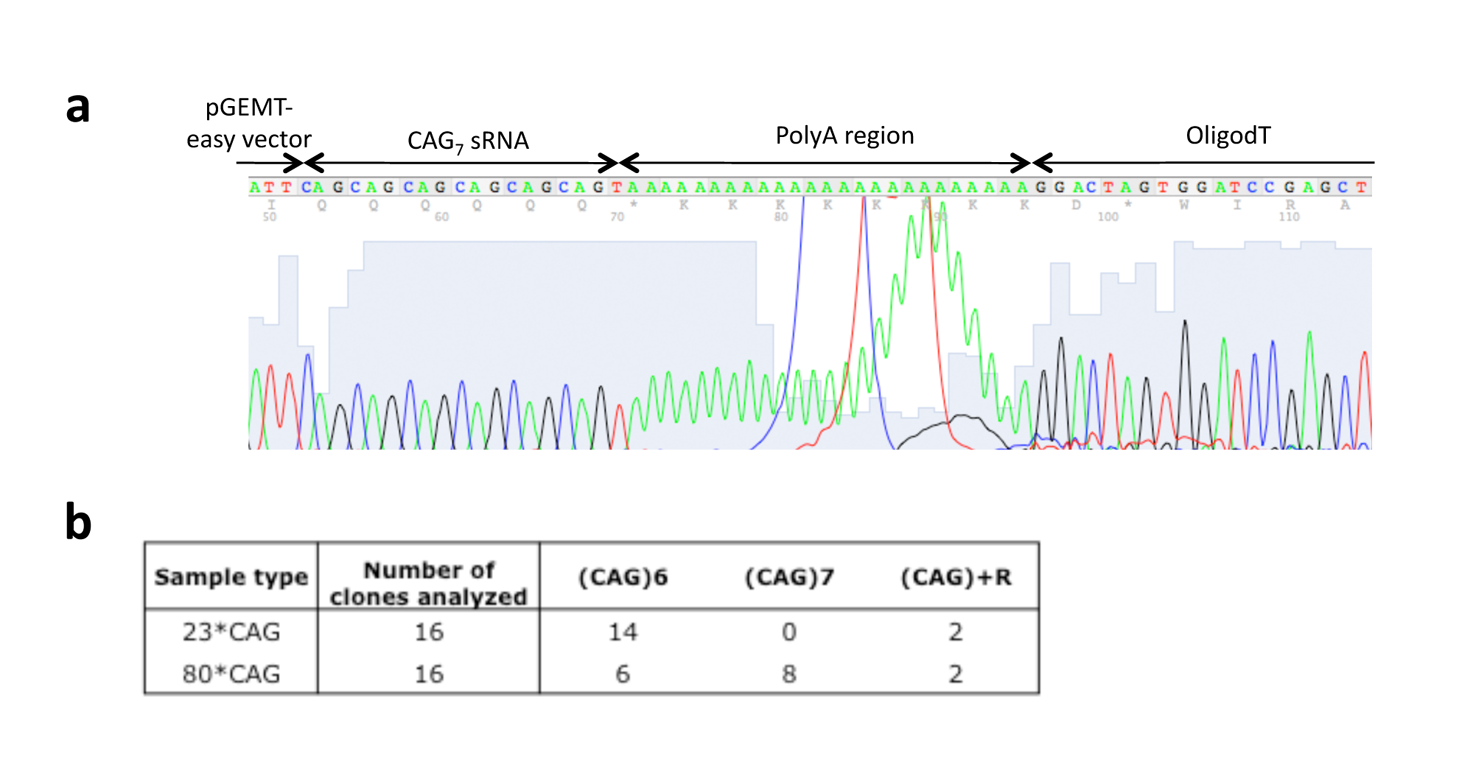

Supplement: Figure S2 — (CAG)7 sRNA are detected in cells expressing 80*CAG HTT. sCAG were detected in cells expressing HTT-e1-expressing vectors by RNA polyadenylation, polyT-based RT and PCR (a detailed description is provided in Methods). PCR products were cloned into pGEMT-easy vector and then sequenced. A. Histogram showing the cloned 21 nt-long (CAG)7 sRNA. Sequences corresponding to the cloning vector, the polyA region and the RT-PCR adapter oligonucleotide are indicated. B. Table shows the type of sequences identified in the different samples and their frequency. (CAG)7 sRNA were only detected in cells expressing 80*CAG HTT (RNA or protein). Sixteen different positive sCAG-cloning colonies were sequenced for each HTT variant. (TIF) [file pgen.1002481.s002.tif]

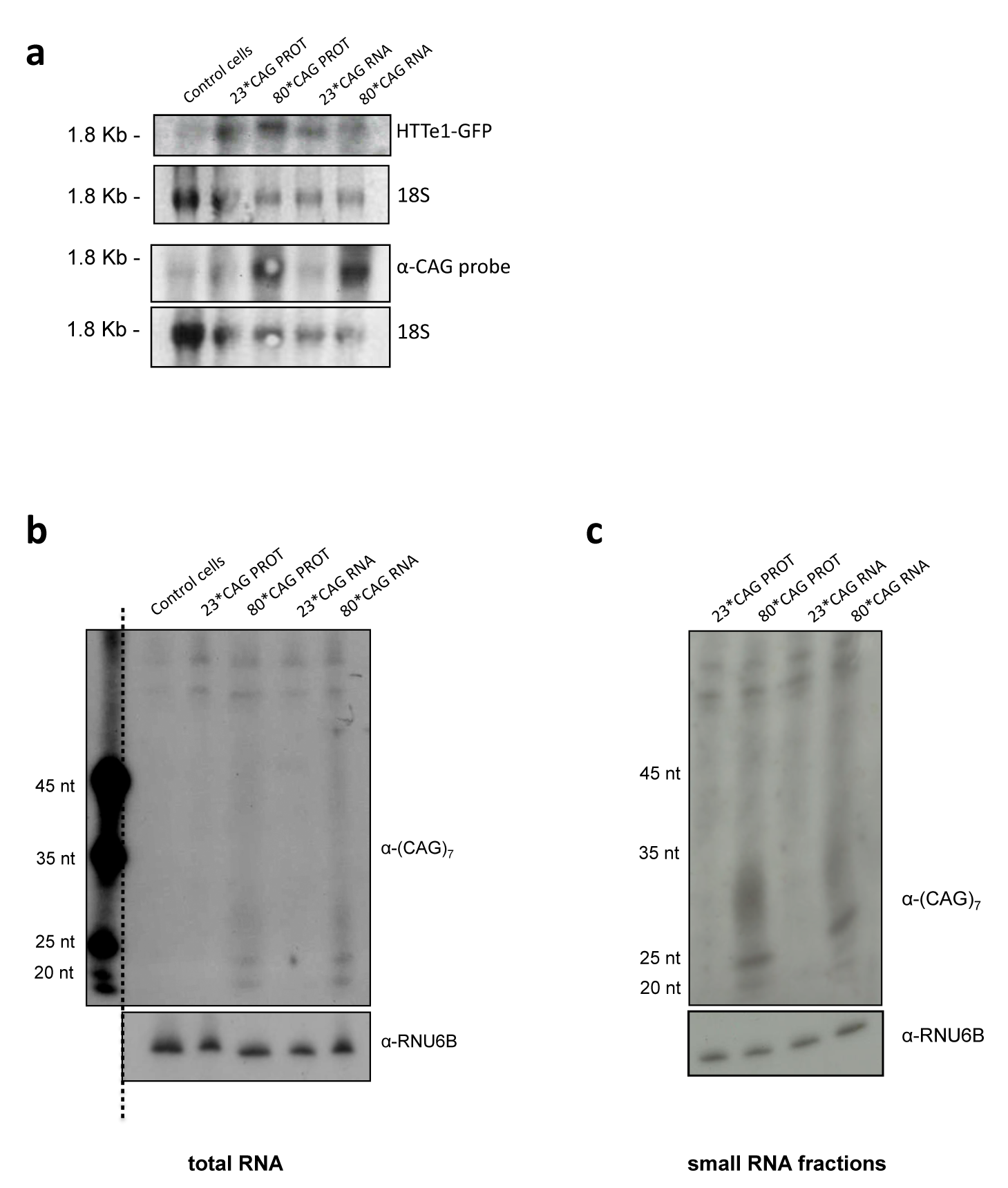

Supplement: Figure S3 — Increased sCAG levels can be detected by Northern blot. Northern blot analysis confirms the increase in sCAG in cells expressing expanded HTT-e1. A. The expression of the different HTT-e1 expression vectors can be detected by α-CAG and α-GFP probes. RNA18S signal was used as loading control. B. CAG-repeated sRNA of around 21 nt long and longer CAG-rich species are produced in cells expressing expanded HTT-e1 RNA. RNU6B sRNA was used as loading control. Thirty µg of total RNA was used per lane. C. sCAG products were also detected in sRNA fraction obtained from cells expressing expanded HTT-e1 (4 µg of RNA<100 nt were subjected to electrophoresis). (TIF) [file pgen.1002481.s003.tif]

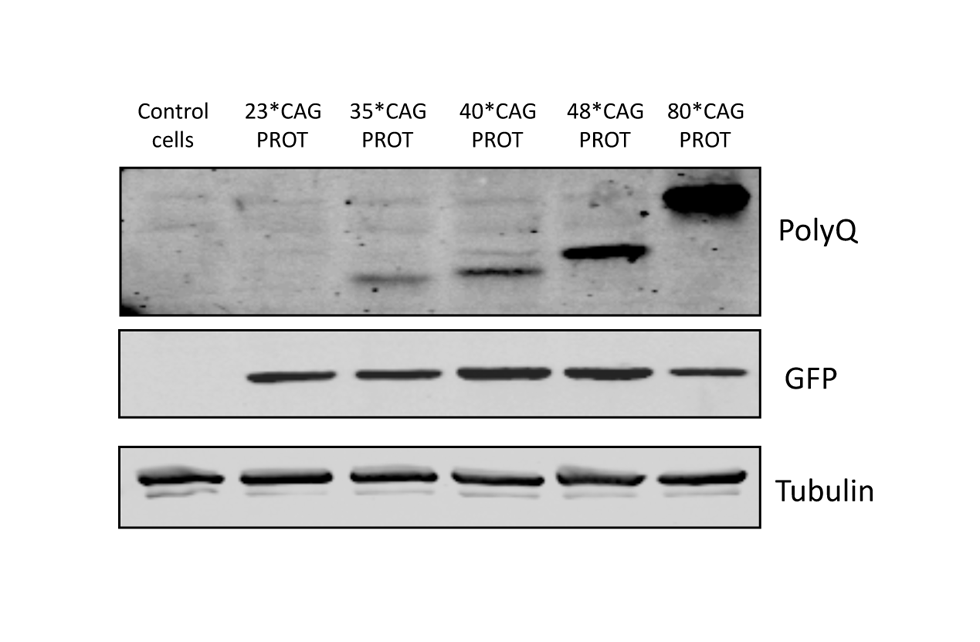

Supplement: Figure S4 — HTT-e1-IRES-GFP vectors are efficiently expressed in SH-SY5Y. Differentiated neuroblastoma cells were transfected with HTT-e1 constructs harbouring 23, 35, 40, 48 or 80 CAG repeats. Western blots shows polyQ and GFP expression. The results are representative of 3 independent experiments. (TIF) [file pgen.1002481.s004.tif]

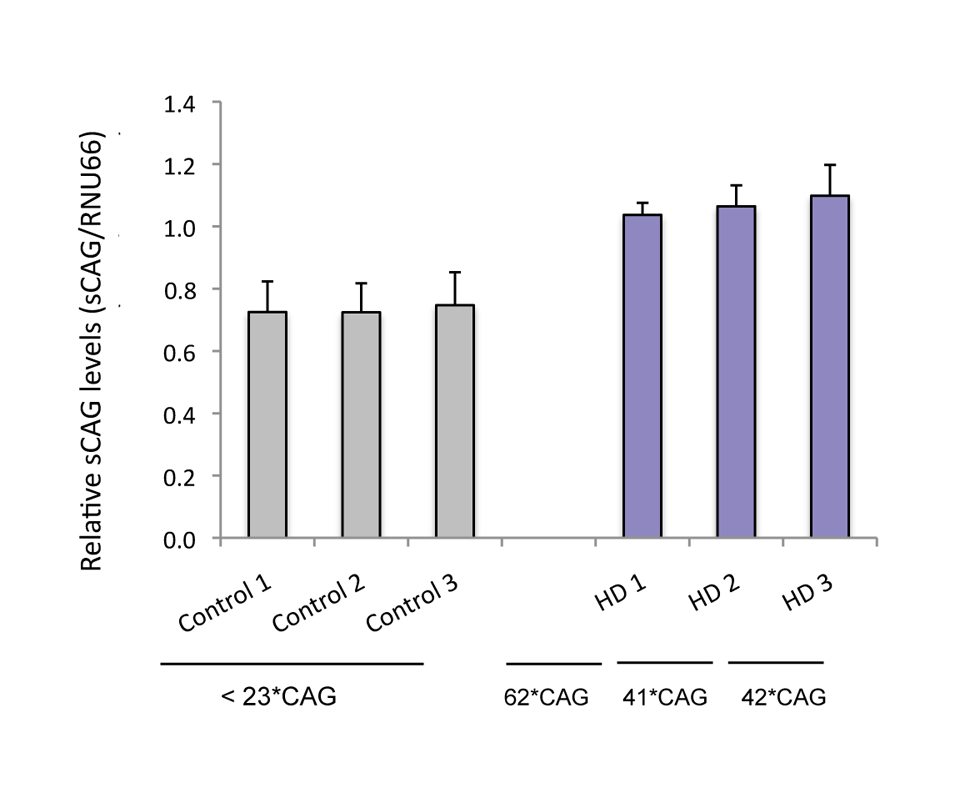

Supplement: Figure S5 — sCAG levels in individual HD brain samples. Graph shows relative sCAG levels in control and HD human brain samples containing different number of CAG repeats. RNU66 was used as the reference sRNA. RT-PCR assays showed that all the HD brain samples analyzed displayed increased sCAG levels compared to control samples. The results are representative of 3 independent experiments performed in triplicate. (TIF) [file pgen.1002481.s005.tif]

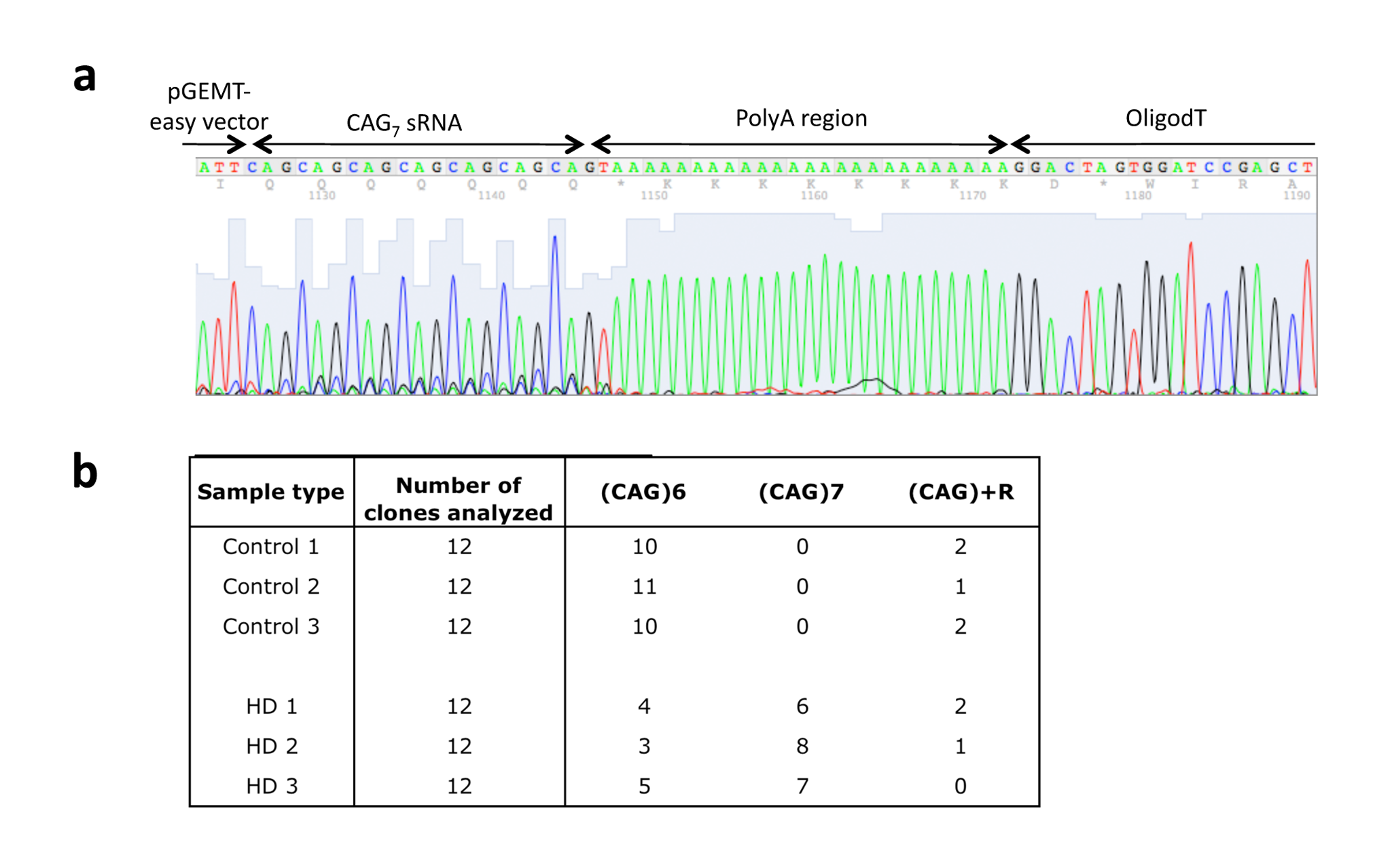

Supplement: Figure S6 — sCAG are detected in HD human frontal cortex. sCAG PCRs from Control and HD frontal cortex were resolved in polyacrylamide gels. Gel fragments corresponding to a size of around 18–24 nt were cut and DNA was isolated and cloned into pGEMT-easy vector. Twelve positive colonies were sequenced for each sample (3 HD-derived brain samples; 3 samples from control subjects). A. Histogram with the sequencing results of HD-derived sample expressing (CAG)7 sRNA. B. Table showing the type of sequences identified in the different samples and their frequency. sRNA constituted by 6*CAG repeats were found in both and HD brains. (CAG)7 sRNA were only found in HD-derived samples. (TIF) [file pgen.1002481.s006.tif]

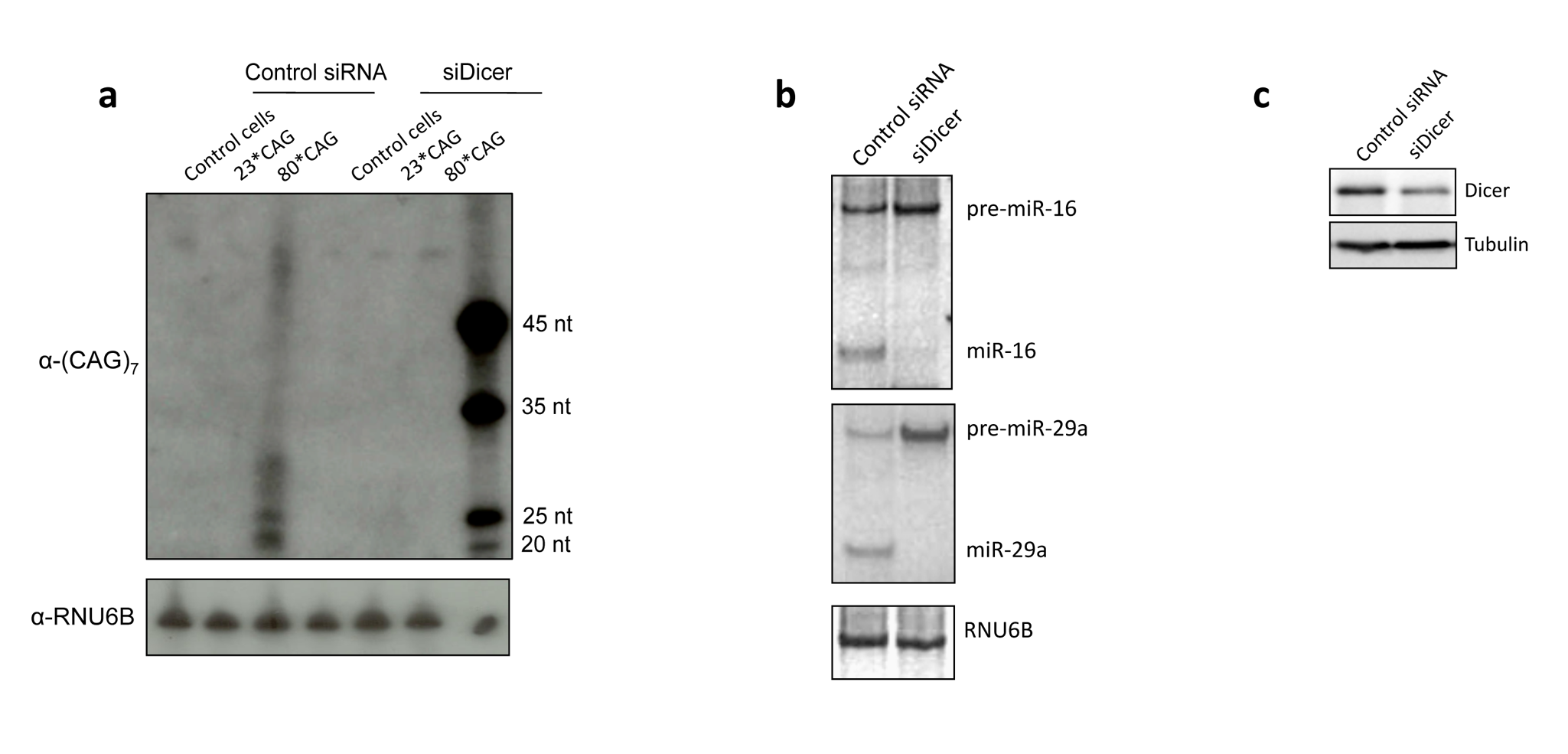

Supplement: Figure S7 — sCAG generation depends on Dicer expression. A. Northern blot confirming that the generation of CAG-repeated sRNAs in cells expressing expanded HTT-e1 is suppressed when cells are depleted of Dicer. RNA6B was used as a loading control. B. Dicing efficiency was determined in normal cells and cells showing low Dicer levels by measuring miRNA maturation. Gels show precursor and mature forms of miR-16 and miR-29a (very abundant and low abundant in SH-SY5Y cells respectively). C. Western blot showing Dicer protein levels in control cells and cells treated with siRNA against Dicer. RNA was isolated 24 hours after transfection with the HTT-e1 expressing vectors. (TIF) [file pgen.1002481.s007.tif]

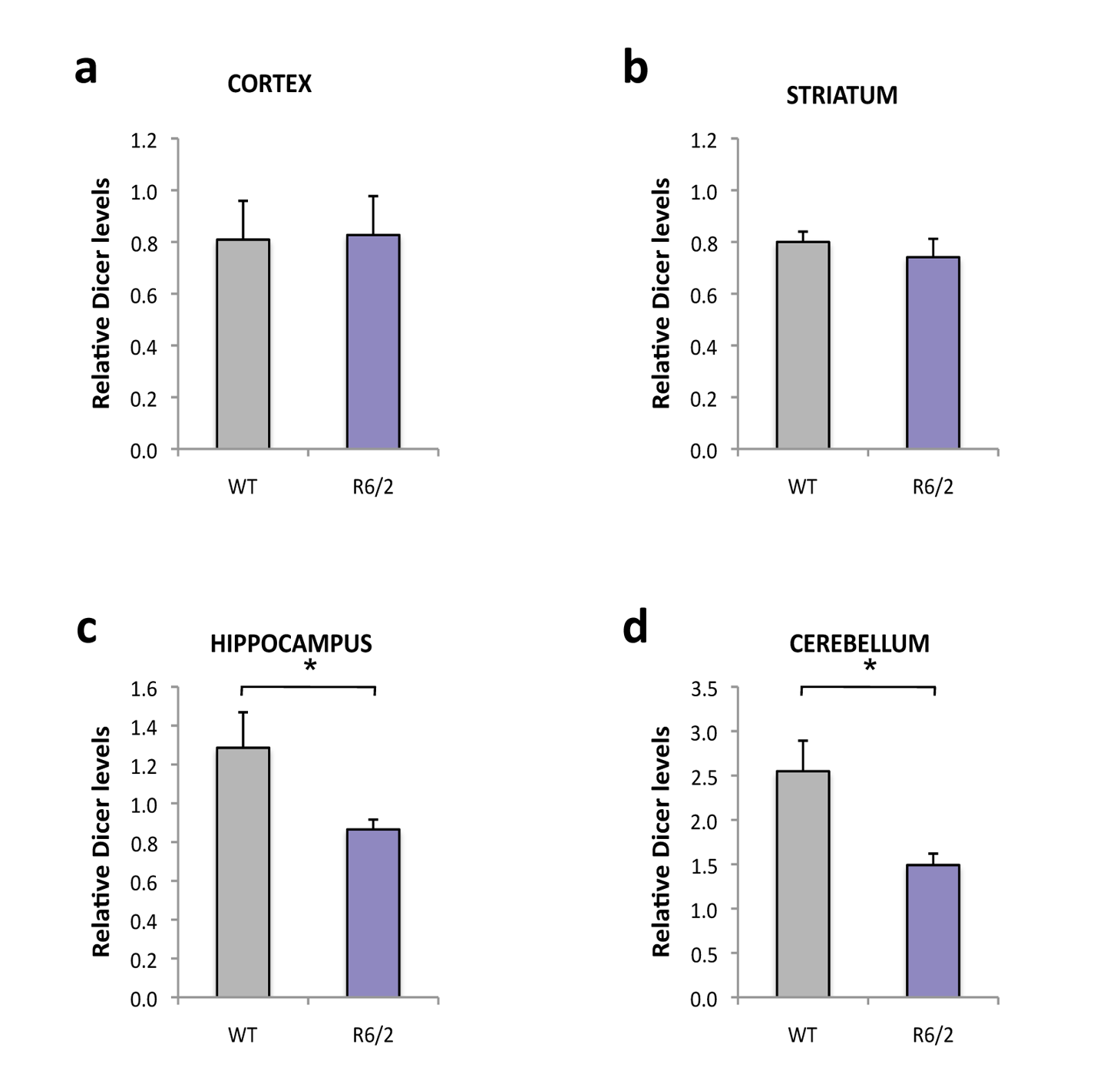

Supplement: Figure S8 — Dicer levels are significantly decreased in the hippocampus and the cerebellum of R6/2 mice. Protein lysates were obtained from striatum, cortex, hippocampus and cerebellum of control and R6/2 mice (8 weeks old). Dicer protein levels were evaluated in four different control mice and five R6/2. Graphs show the densitometry determination of Dicer levels vs Tubulin for each specific brain area. Results demonstrate that Dicer levels are decreased in hippocampus and cerebellum of R6/2 compared to its levels in control mice. Results represent the mean arbitrary optical density change ± SEM (n = 3; *p<0.05). (TIF) [file pgen.1002481.s008.tif]

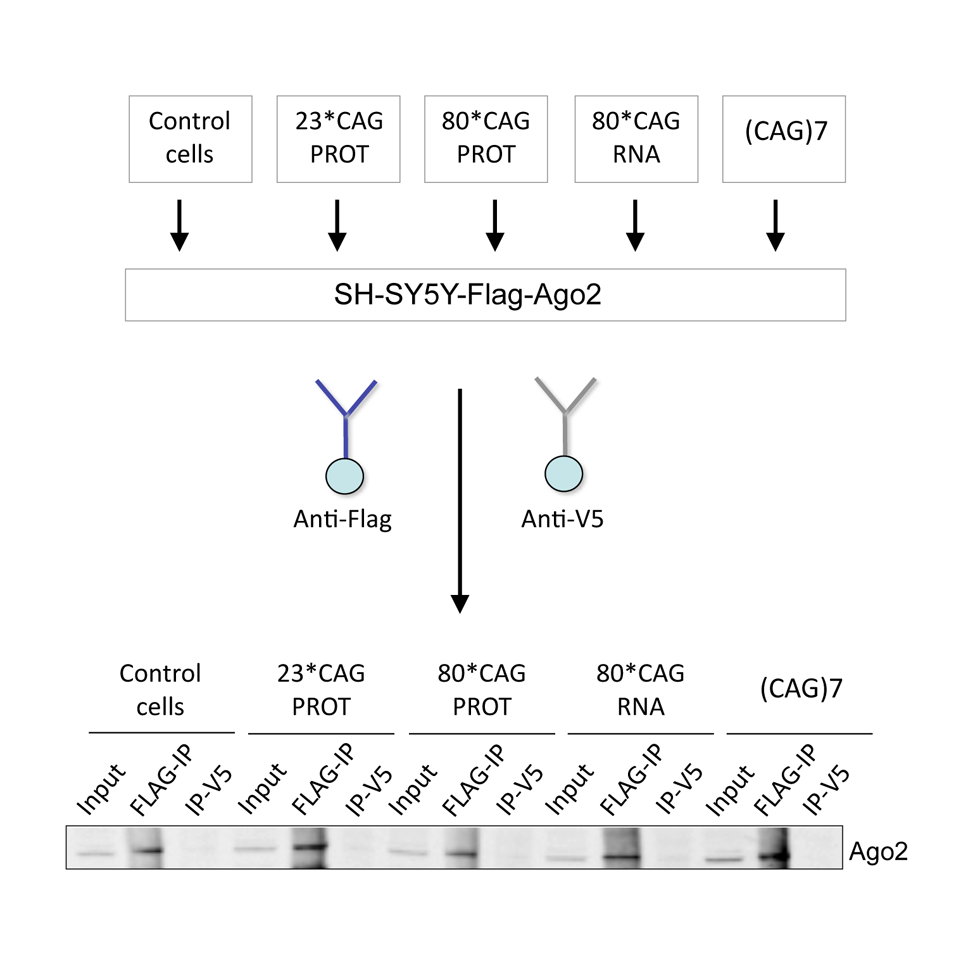

Supplement: Figure S9 — Experimental design to detect CAG-repeated small RNAs in Ago2 complexes. SH-SY5Y cells stably expressing mild levels of the Flag-Ago2 fusion protein were transfected with HTT-expressing vectors (protein or RNA expressing constructs). Immunoprecipitation (IP) experiments were performed using an anti-Flag tag antibody or an anti-V5 tag antibody as negative control for Ago2 binding. Western blot analysis shows that Ago2 protein was only immunoprecipitated by the use of Flag antibodies. These immunoprecipitates were used for the detection of sCAG (Figure 4D). (TIF) [file pgen.1002481.s009.tif]

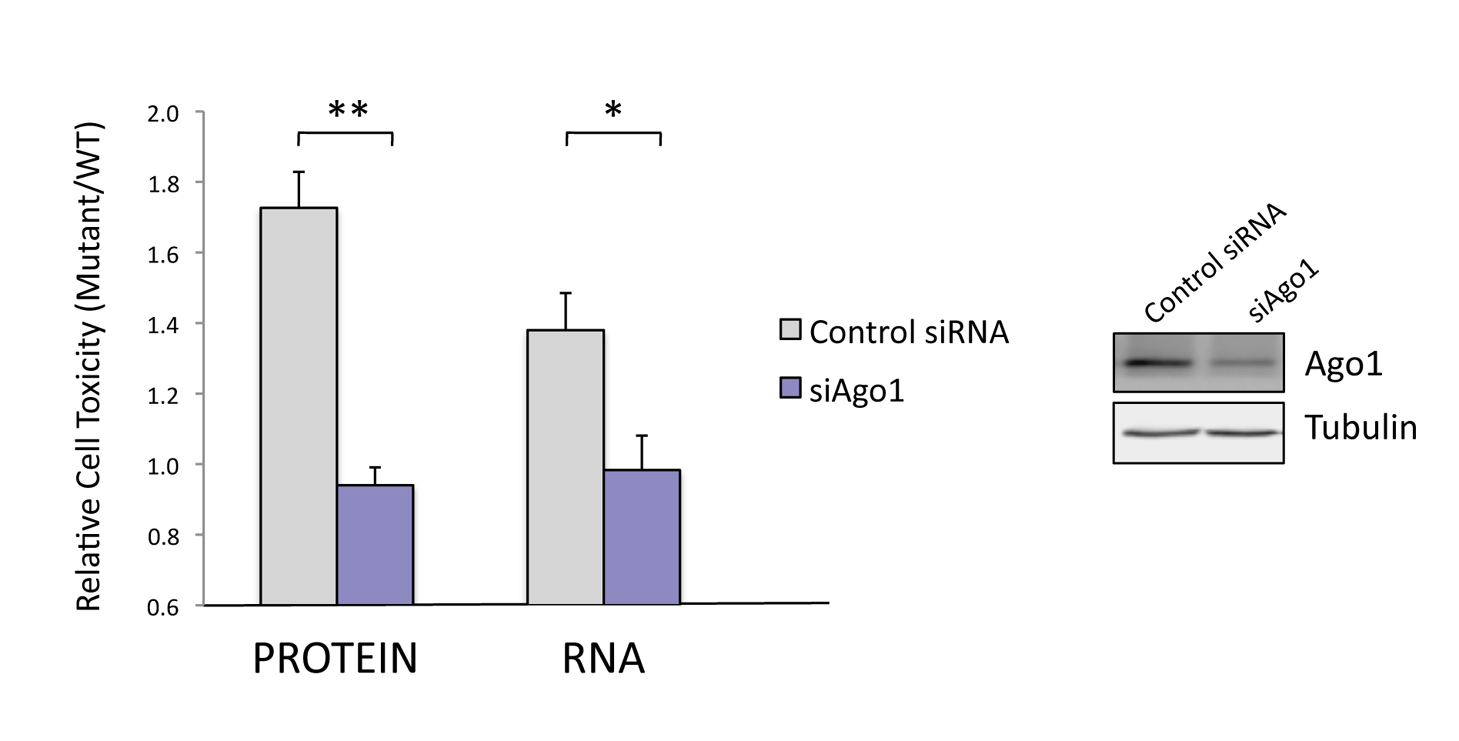

Supplement: Figure S10 — Ago1 activity contributes to HTT RNA toxicity. SH-SY5Y cells were transfected with siRNAs specific for Ago1 (siAgo1) or scrambled siRNAs as control. Cells were co-transfected 48 hours later with the combination HTT-e1/siAgo1 or HTT-e1/control siRNA. A. LDH cytotoxicity assay shows that the detrimental effect of CAG-expanded HTT RNA is dramatically reduced when cells are depleted of Ago1 (n = 3; *p<0,05 **p<0,01). Values are referred to the control cells lacking HTT expression. Values represent mean of the ratio: expanded-HTT RNA toxicity vs non-expanded-HTT RNA toxicity ± SEM for each experimental condition. (TIF) [file pgen.1002481.s010.tif]

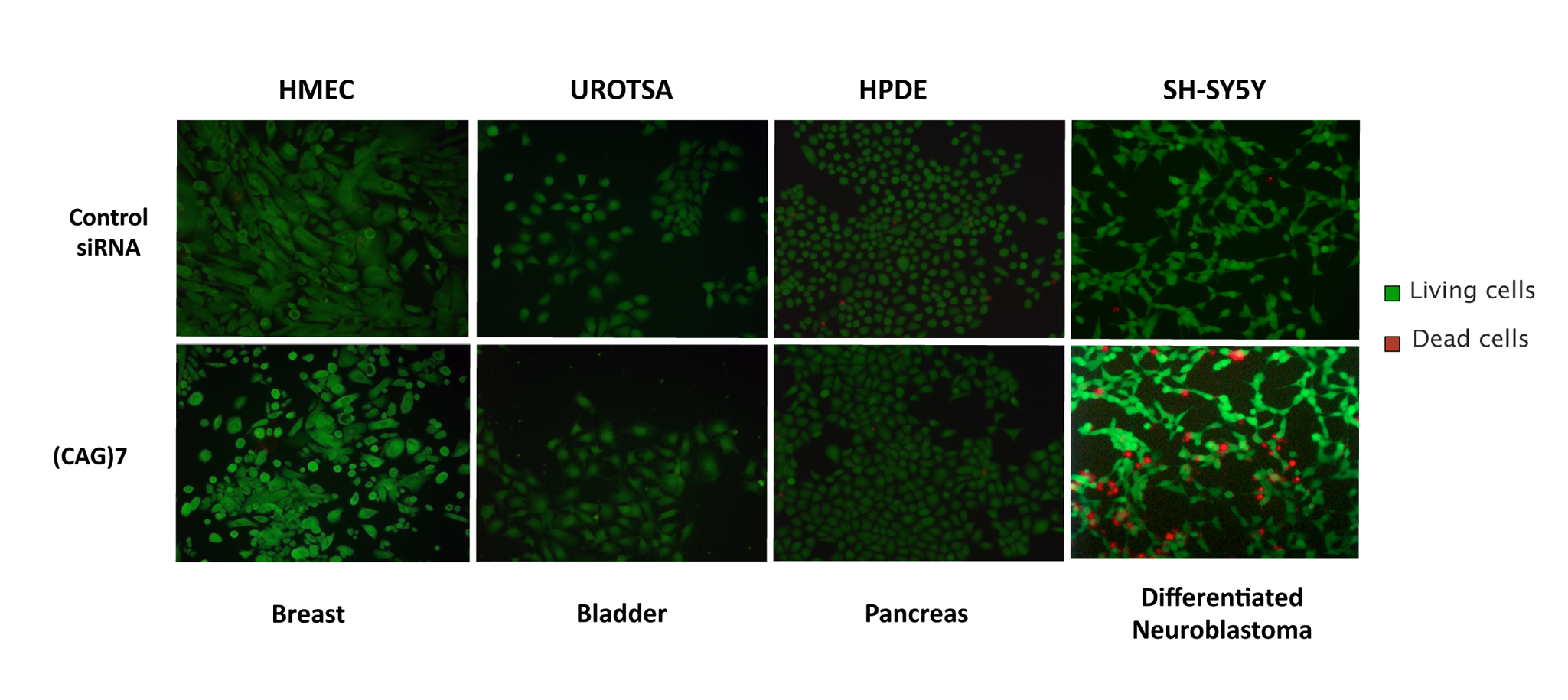

Supplement: Figure S11 — Cell-type dependent cytotoxic effect of (CAG)7 siRNAs. (CAG)7 siRNA was transfected into HMEC, UROTSA and HPDE non-neural human cells. Differentiated SH-SY5Y cells were used as a post-mitotic neuronal cell model. (CAG)7 siRNA exert a cell-type dependent toxic effect, preferentially affecting neuronal viability. Fluorescein diacetate staining (green) labels living cells. Propidium iodide staining (red) specifically labels dead cells. Stainings were performed 36 hours after transfection (n = 3, each experiment performed in triplicate). (TIF) [file pgen.1002481.s011.tif]

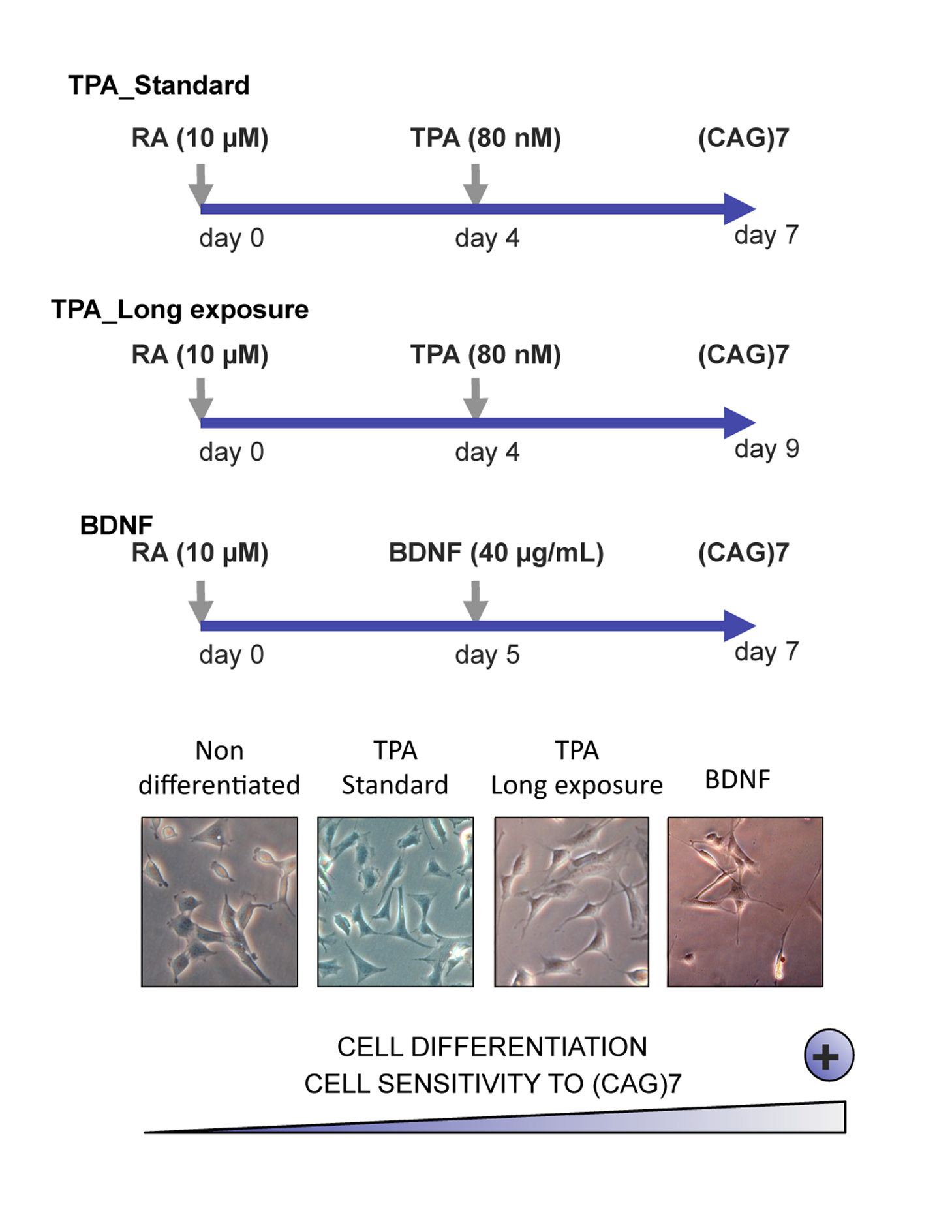

Supplement: Figure S12 — Neuronal differentiation protocols used in SH-SY5Y cells. Schematic description of the different neuronal differentiation protocols used to characterize the toxic effect of (CAG)7 siRNA (Figure 5B). Pictures show the morphological changes induced after each differentiation condition. (TIF) [file pgen.1002481.s012.tif]

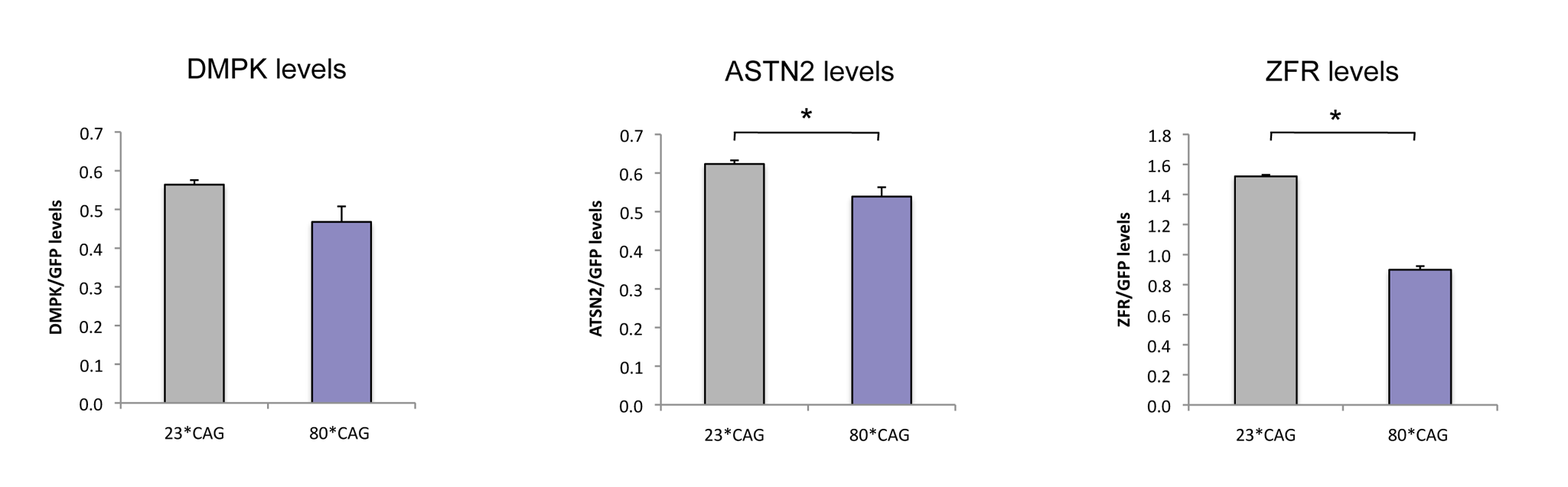

Supplement: Figure S13 — Expanded HTT-e1 RNA silences CUG rich genes. Total RNA was isolated from cells expressing the different HTT-e1 expressing vectors 24 hours after transfection. qRT-PCR revealed that expanded HTT-e1 (protein and RNA) can suppressed endogenous levels of CUG rich transcripts (DMPK, ASTN2 and ZFR). The percentage of suppression differs among the transcripts analyzed. (n = 4; *p<0,05, experiments performed in triplicates). (TIF) [file pgen.1002481.s013.tif]
